# Exome sequencing of primary breast cancers with paired metastatic lesions reveals metastasis-enriched mutations in the A-kinase anchoring protein family (AKAPs)

**DOI:** 10.1186/s12885-018-4021-6

**Published:** 2018-02-12

**Authors:** Una Kjällquist, Rikard Erlandsson, Nicholas P. Tobin, Amjad Alkodsi, Ikram Ullah, Gustav Stålhammar, Eva Karlsson, Thomas Hatschek, Johan Hartman, Sten Linnarsson, Jonas Bergh

**Affiliations:** 1 Department of Oncology and Pathology, Cancer Center Karolinska, Karolinska Institute and University Hospital, Stockholm, Sweden; 2 Department of Medical Biochemistry and Biophysics, Karolinska Institutet, Stockholm, Sweden; 3 Research Programs Unit, Genome-Scale Biology and Medicum, University of Helsinki, Helsinki, Finland

**Keywords:** Breast cancer, Metastasis, Exome sequencing, Somatic mutations, AKAP, A-kinase anchoring proteins

## Abstract

**Background:**

Tumor heterogeneity in breast cancer tumors is today widely recognized. Most of the available knowledge in genetic variation however, relates to the primary tumor while metastatic lesions are much less studied. Many studies have revealed marked alterations of standard prognostic and predictive factors during tumor progression. Characterization of paired primary- and metastatic tissues should therefore be fundamental in order to understand mechanisms of tumor progression, clonal relationship to tumor evolution as well as the therapeutic aspects of systemic disease.

**Methods:**

We performed full exome sequencing of primary breast cancers and their metastases in a cohort of ten patients and further confirmed our findings in an additional cohort of 20 patients with paired primary and metastatic tumors. Furthermore, we used gene expression from the metastatic lesions and a primary breast cancer data set to study the gene expression of the AKAP gene family.

**Results:**

We report that somatic mutations in A-kinase anchoring proteins are enriched in metastatic lesions. The frequency of mutation in the AKAP gene family was 10% in the primary tumors and 40% in metastatic lesions. Several copy number variations, including deletions in regions containing AKAP genes were detected and showed consistent patterns in both investigated cohorts. In a second cohort containing 20 patients with paired primary and metastatic lesions, AKAP mutations showed an increasing variant allele frequency after multiple relapses. Furthermore, gene expression profiles from the metastatic lesions (*n* = 120) revealed differential expression patterns of AKAPs relative to the tumor PAM50 intrinsic subtype, which were most apparent in the basal-like subtype. This pattern was confirmed in primary tumors from TCGA (*n* = 522) and in a third independent cohort (*n* = 182).

**Conclusion:**

Several studies from primary cancers have reported individual AKAP genes to be associated with cancer risk and metastatic relapses as well as direct involvement in cellular invasion and migration processes. Our findings reveal an enrichment of mutations in AKAP genes in metastatic breast cancers and suggest the involvement of AKAPs in the metastatic process. In addition, we report an AKAP gene expression pattern that consistently follows the tumor intrinsic subtype, further suggesting AKAP family members as relevant players in breast cancer biology.

**Electronic supplementary material:**

The online version of this article (10.1186/s12885-018-4021-6) contains supplementary material, which is available to authorized users.

## Background

The mutational landscape of primary breast cancer tumors has been extensively studied in recent years and a large number of exomes and full genomes have become available [1–3]. Most studies to date have focused on the primary breast tumor whilst mutational profiles of metastatic lesions and their relationship to the primary tumor have largely been lacking. This has important clinical implications as altered receptor status in the metastatic lesion has been shown to occur at high rates ranging from 14.5–40% for ER 40% for PgR and 0–37% for HER2-receptors during cancer progression [4–6] and is additionally affected by adjuvant therapy with major implications for management of the metastatic disease. The genetic evolution and accumulation of genetic aberrations in metastatic malignancies has been described in [7–13]. Most importantly, accumulation of activating estrogen receptor 1 (ESR1) mutations in metastatic lesions has demonstrated a mechanism of acquired hormone resistance in metastatic breast cancer [14–16].

In order to characterize the mutational landscape in primary tumors and metastases, we performed exome sequencing in ten patients for which paired samples were available. We found a pronounced heterogeneity both between and within patients with a general enrichment in number of somatic mutations in metastatic tissues. As a consequence of finding a number of both silent and nonsilent mutations in the *AKAP9* gene, one of the recurrent cancer genes in the COSMIC data base, we investigated all 14 AKAP (A-kinase anchoring proteins) family members and found somatic mutations in seven of them, the majority of which were in metastatic lesions only. In addition, several copy number changes were present in AKAP loci, mostly deletions. The different members of the A-kinase anchoring proteins (AKAPs) directs and orchestrates the activity of Protein Kinase A (PKA), which is an important factor in cell motility and proliferation [17]. In line with the cellular function of PKA, several of the AKAP members have been associated with cancer development and metastatic spread. TCGA data confirms the presence of AKAP somatic point mutations in primary breast cancer with a prevalence of 7% and with a higher rate including copy number variations.

## Methods

### Patient population and ethics

Ten patients representing different clinical subtypes and metastatic lesion sites were included in the study (Table 1). Patients P1 to P7 were part of the Swedish multicenter trial TEX, ClinicalTrials.gov identifier NCT01433614 that enrolled 287 patients with loco-regional or distant breast cancer relapse from 2002 until 2007 [18]. Patients P8 to P10 were part of a prospective biopsy study at Radiumhemmet Karolinska Hospital during 2007–2010. The information on primary and relapse ER, PR and HER2 status were retrospectively collected from pathology reports and reassessed by IHC/ICC. All patients were given informed consent.

**Table 1:** Clinical information cohort 1

Patient ID	Sample		PAM50 subtype	ER	PR	HER2	HER2 CNV (exome data)	AdjCT	AdjHT	AdjRT	DFI	Relapse site	Relapse to death
P1	*Primary Tumor* *Metastasis*	3	*NA* Luminal B	+ -	+ -	- -	HER2- HER2-	+	+	+	3.3	liver	3.2
P2	*Primary Tumor* *Metastasis*	3	*NA* Basal like	- *NA*	- *NA*	- *NA*	HER2- HER2-	+	–	+	1.4	lymph	4.9
P3	*Primary Tumor* *Metastasis*	3	*Na* *Luminal B*	- -	+ -	- *NA*	HER2- HER2-	–	–	+	4.1	liver	1.5
P4	*Primary Tumor* *Metastasis*	3	*NA* Basal like	- *NA*	- *NA*	*NA* *NA*	HER2+ (CN 3.5) HER2+ (CN 3.5)	+	+	+	8.1	lung	6.0
P5	*Primary Tumor* *Metastasis*	N/A	*NA* Luminal A	+ +	- -	- *NA*	HER2- HER2+ (CN 2.5)	–	+	+	3.5	liver	5.7
P6	*Primary Tumor* *Metastasis*	3	*NA* HER2 enriched	+ +	+ -	*NA* -	HER2- HER2-	+	+	+	2.6	lymph	1.1
P7	*Primary Tumor* *Metastasis*	2	*NA* Normal like	+ +	+ -	*NA* -	HER2- HER2-	+	+	+	4.8	skin	0.8
P8	*Primary Tumor* *Metastasis*		*NA* *NA*	+ +	+ +	- -	HER2+ (CN 2.4) HER2- (CN 0.8)	+	+	+	*5* ^a^	liver	0.7
P9	*Primary Tumor* *Metastasis*		*NA* *NA*	- -	- -	*NA* -	HER2- HER2-	+	–	+	*11* ^a^	breast	*NA*
P10	*Primary Tumor* *Metastasis*		*NA* *NA*	+ +	+ +	- -	HER2- HER2-	+	+	+	*4* ^a^	bone	0.4

Receptor status by IHC (immunohistochemistry)

*ER* estrogen receptor, *PR* progesteron, *HER2* Her2 receptor status, *HER2 CNV* amplification of Her2 derived from exome data, *AdjCT* adjuvant chemotherapy, *AdjHT* adjuvant hormone therapy, *AdjRT* adjuvant radiotherapy, *DFI* disease free interval (months); Relapse to death (months)

^a^approximated as time from primary tumor surgery date to relapse diagnosis date

Patients for cohort 2 (Additional file 1: Table S1) were identified using search criteria in the digital patient record system which allowed collection of formalin-fixated paraffin-embedded (FFPE) material from primary breast cancer, local recurrence, axillary- and distant metastases as well as clinical information as described in Ullah et al. (submitted). Samples were obtained only after approval by the Ethical committee at Karolinska Institutet, Stockholm. Samples were further investigated and assessed by a certified surgical pathologist. All studies were approved by the Regional Ethical Review Board in Stockholm, Sweden.

### Cohort 1: DNA extraction and exome capture

DNA was extracted from fresh frozen fine needle aspiration (fnac) biopsies (metastases), fresh frozen surgical material (primary tumors) and blood (germline control), concentrations were determined using Qubit dsDNA HS assay (Life Technologies). Due to low concentrations obtained from aspirations, we used WGA in order to meet the required amounts of DNA needed for exome enrichment. All paired samples including germline DNA were subjected to WGA using GenomiPhi V2 DNA Amplification Kit (GE Healthcare) according to manufacturer’s instructions, a summary of DNA concentrations can be found in Additional file 2: Table S2. The fragment length after ultrasonic DNA fragmentation by Covaris (LGC Genomics) was measured using 2200 TapeStation Genomic DNA screen tape (Agilent Technologies), amplified DNA did not differ from non-amplified DNA (data not shown).

Exome capture from amplified tumor and normal DNA was performed using Sure Select Human All Exon V4 (Agilent Technologies) according to the SureSelect^XT^ Target Enrichment System for Illumina Paired-End Sequencing manual version 1.3. Sample preparation, hybridization and post-hybridization amplification were performed according to the manufacturers instructions, with five cycles of PCR in amplification of adaptor-ligated library, and 12 cycles in the post-hybridization captured library amplification step. The quality of final libraries was evaluated using TapeStation High Sensitivity DNA kit (Agilent) and the libraries were quantified using KAPA SYBR® FAST ABI Prism qPCR Kit (KAPA Biosystems). Enriched exome fragments were pooled and paired-end sequenced on a HiSeq 2000 platform (Illumina).

### Cohort 1: Variant detection

Demultiplexing, alignment and variant detection were carried out using the Casava 1.8.2 pipeline from Illumina with default parameters and duplicate removal. Samtools v0.1.9 (r783) was used to create mpilups from the bam files and variant identification was performed using the VarScan (v2.3.2) [19] somatic function with default parameters, but a minimum variant frequency of 5% and a somatic *P* value of 0.2. The VarScan-identified positions were examined using the Ensembl variant prediction tool (v 2.6 and database ver 69) and known SNPs were labeled using the SnpEff database (SnpSift version 1.9c). The VarScan, Variant effect prediction, SnpEff and mpileup base count outputs were joined on position using in-house scripts to facilitate downstream filtering and comparison. The output data was filtered by retaining only those positions with variant frequency in germline sample < 1%, variant reads in tumor ≥ 5, and total coverage > 30. Average coverage per exome was 47.3 Mb (range 18.8–51.1 M) (Additional file 3: Figure S2a). SNVs, CNVs and LOH were called by comparative analysis of sequence variants between tumor and germline samples. To account for potential false positive mutation calls, the output data was manually filtered and positions with variant frequency in germline sample < 1%, variant reads in tumor < 5 and a total coverage of < 30 reads were excluded, leaving only exome wide mutations with an VAF > 16%.

To focus the analysis, a set of 505 recurrently mutated genes was used to select somatic variant positions of interest. For these genes, all variant positions occurring in the pairs of primary and metastatic lesions were selected, including those with less than five tumor variant reads, not to exclude true shared low frequency mutations. The selected positions were manually investigated, visualizing the unfiltered reads in Integrative Genomics Viewer (Broad Institute) with respect to alignment quality, base quality of variant base, read direction and total coverage on the position. Variant positions with the following conditions were manually removed from the data set: variant reads in normal sample, poor alignment or highly variable sequences and variant reads in one direction only.

### Cohort 2: DNA extraction and exome capture

We used additional exome sequences available from paired primary and metastatic samples from 20 patients to test our findings from cohort 1 (Additional file 1: Table S1). DNA isolation, exome capture and sequencing was performed by SeqWright Genomic Services (GE Healthcare, Houston, USA). DNA was isolated from serial thick sections of FFPE tissues using a QIAamp DNA FFPE Tissue Kit (Qiagen, CA, USA) a summary of DNA concentrations can be found in Additional file 2: Table S2. DNA from normal axillary lymph nodes FFPE tissues was used as germline controls. In all cases we followed the manufacturer’s recommended protocol. Exome capture was performed using Sure Select XT2 Human All ExonV5 (Agilent Technologies) according to manufacturers instructions. Paired end sequencing was performed on Illumina HiSeq 2500.

### Cohort 2: Variant detection

Raw sequencing reads were quality and adapter trimmed using trim_galore, where the first 13 bases of Illumina adapter were used and stringency parameter was set to 2. Reads having lengths less than 70 after trimming were filtered out with their paired mates. Trimmed reads were aligned to the human reference genome build hg19 using bwa-mem with default parameters. The aligned reads were marked for duplicates by Picard (25%), realigned around known indels and base-quality recalibrated by GATK. Somatic single nucleotide variants (SNVs) were detected by Mutect [20] with the high-confidence mode. Copy number alterations were detected by ADTEx [21]. All pipelines and analyses were run using Anduril [22], a workflow framework for scientific data analysis. Mean coverage per sample is given in (Additional file 3: Figure S2b).

### CNV calculations

Copy number variations (CNVs) were determined by calculating the normalized read coverage at each SNP position for paired tumor and germline samples, then taking the log_2_ of the ratio of these numbers and recentering to zero. In Additional file 4: Figure S1a-b, these measures were plotted as a moving average across ten adjacent SNPs.

### LOH calculations

Estimating LOH fraction and tumor content using a Beta-Normal mixture model Additional file 5: Figure S3a-b. Histograms show the distribution of major allele frequencies (range: 50–100%) and red curves show the mixture model estimated from the data. The set of SNPs called in the germline sample was considered in the tumor sample (primary and metastasis independently). When genomes contain regions of LOH, the distribution will be bimodal, as illustrated in the inset (top right). The first component (closer to 50%; red in inset) represents heterozygous SNPs in regions without LOH and was modeled as a normal distribution with a mean close to 50% in a perfect sample, but will increase towards 100% as allelic dropout increases.

The second component (closer to 100%; blue in inset) represents homozygous SNPs in regions with LOH, was modeled as a beta distribution with two parameters (the mean of this component should be close to 100% and increase as LOH fraction increases and decrease as with non-cancer cell contamination. The mixture distribution thus had four free parameters plus the mixture proportion, the latter representing the estimated LOH fraction of the sample. Fitting this mixture to the observations yielded estimates for LOH fraction, tumor fraction and allelic dropout rate for each sample (Additional file 5: Figure S3). Model fitting was performed using the *EstimatedDistribution* function of Mathematica 9.0 (Wolfram Research Inc.).

### Microarray

Microarray experiment was performed as follows; total RNA from frozen metastatic tumors was extracted using the Qiagen RNeasy Mini Kit (Qiagen, Germany). All patient tumor samples were profiled using NuGEN amplification protocol and hybridized using the HRSTA-2.0 custom human Affymetrix array GPL10379. The microarray gene expression analyses were performed in R using the aroma.affymetrix package. The PAM50 intrinsic subtypes (PAM50) for cohort 1 relapses have been previously published [23] and publicly available GSE56493. The case-control cohort have been previously published [24] GSE48091. The normalized expression array and intrinsic subtype calls for the TCGA data were taken from the original manuscript [1].

### Control experiment

To control for amplification-induced artefacts i.e. false positive and false negative mutation rates, we designed a control experiment as follows: Genomic DNA from a healthy individual was extracted from whole blood using the PAXgene Blood DNA kit (Qiagen). The DNA was of good quality (> 58 kB fragment length) and of high concentration (> 300 ng/uL). This DNA was diluted 1:100 and 1:1000 and 6 ng respectively 0.6 ng was subjected to WGA, exome enriched, sequenced and analyzed as described above using an unamplified sample as ‘germline’ control, (Additional file 6: Table S3).

## Results

We first analyzed somatic mutations in a set of 505 previously recognized recurrent cancer genes from COSMIC census database and The Cancer Genome Atlas [1] (henceforth called ‘recurrent cancer genes’) where an enrichment for true driver mutations may be expected. We detected a total of 654 somatic mutations, of which 301 were nonsynonymous, distributed over 190 different genes. Of nonsynonymous mutations, an average of 43 and 47% were predicted to be deleterious and tolerated, respectively.

### The number of somatic mutations was significantly different between primary tumor and metastatic lesion

**Fig. 1:**
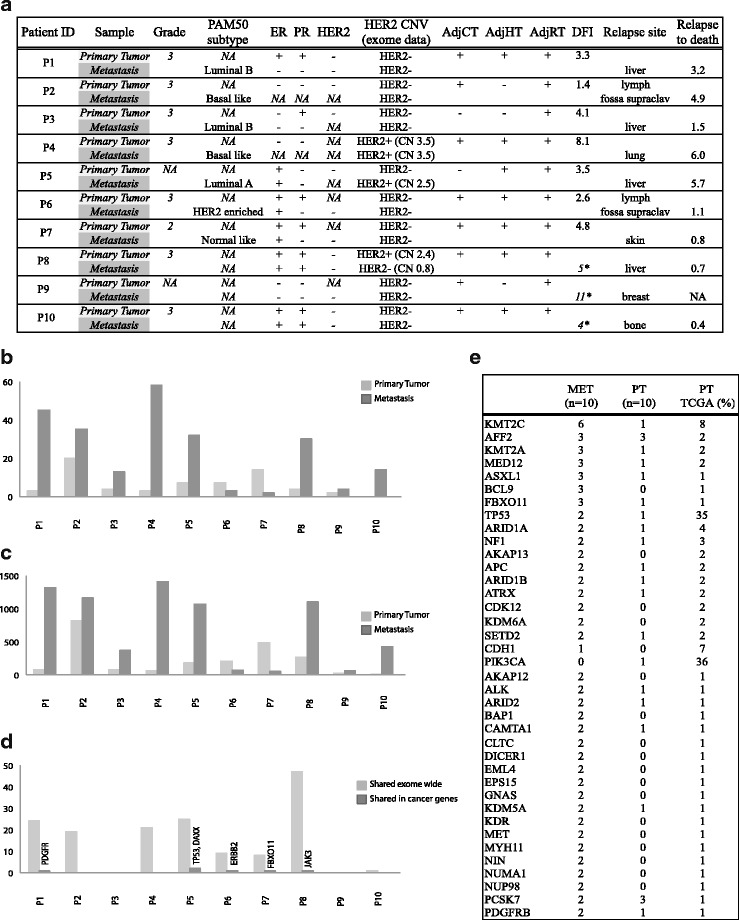
**a** Number of nonsynonymous somatic mutations in recurrently mutated cancer genes. Mutations in genes previously reported mutated in different cancers (COSMIC). The primary tumors and metastases carried an average of 6 (range: 0–20) and 24 (range: 2–58) nonsynonymous mutations respectively (*p* < 0.001; Student’s t-test). **b** Number of nonsynonymous somatic mutations in all captured exomic regions. Primary tumors showed an average of 222 nonsynonymous mutations (range: 11–825) whereas the mutational load in metastases was significantly greater in metastases (average: 706, range: 51–1411; *p* < 0.05; Student’s t-test). **c** Number of somatic mutations shared in primary tumor and its corresponding metastatic lesion. All variant positions occurring in the pairs of primary and metastatic lesions were selected including those with less than 5 tumor variant reads. The positions were manually investigated as described in methods section

The mutational load was significantly greater in metastases, both in recurrently mutated genes, (average: 6, range: 0–20 vs average: 24, range: 2–58; *p* < 0.001; Student’s t test) (Fig. 1a) and in all exomic regions (average: 222, range: 11–825 vs average: 706, range: 51–1411; *p* < 0.05; Student’s t-test) (Fig. 1b). In eight of the patients, the metastatic lesions showed a higher number of somatic point mutations than the primary tumor (range: 2–19 fold higher). Interestingly, in two of the patients (patients P6 and P7) the relationship was inverted, with fewer mutations in recurrently mutated genes in the metastatic tissue (Fig. 1a; 2.3 and 7 fold fewer) and (Fig. 1b; 3 and 10 fold fewer). The lower number of mutations in these metastases coincided with a reduced level of CNV and LOH in these two patients (Additional file 4: Figure S1b). This unexpected pattern was also seen in five of 20 patients in cohort 2 (data not shown). The number of shared mutations varied between 0 and 47 (average 15.4) in whole exomes and included potential driver genes *PDGFR, TP53, DAXX, ERBB2, FBOXO11* and *JAK3* (Fig. 1c). Despite the small cohort we found several mutations in previously described genes, frequently mutated in breast cancer [1–3] (Table 2). Importantly, no recurrent cancer genes were found mutated in the control experiment.

**Table 2:** Most frequently mutated genes in cohort1 sorted by occurrence in metastases

	Metastasis *n* = 10 (%)	Primary tumors *n* = 10 (%)	Primary tumors TCGA (%)
KMT2C	6 *(60)*	1 *(10)*	8
AFF2	3 *(30)*	3 *(30)*	2
KMT2A	3 *(30)*	1 *(10)*	2
MED12	3 *(30)*	1 *(10)*	2
ASXL1	3 *(30)*	1 *(10)*	1
FBXO11	3 *(30)*	0	1
TP53	2 *(20)*	1 *(10)*	35
ARID1A	2 *(20)*	1 *(10)*	4
NF1	2 *(20)*	1 *(10)*	3
AKAP13	2 *(20)*	0	2
APC	2 *(20)*	1 *(10)*	2
ARID1B	2 *(20)*	1 *(10)*	2
ATRX	2 *(20)*	1 *(10)*	2
CDK12	2 *(20)*	0	2
KDM6A	2 *(20)*	0	2
CDH1	1 *(10)*	0	7
PIK3CA	0	1 *(10)*	36
AKAP12	2 *(20)*	0	1
ALK	2 *(20)*	1 *(10)*	1
ARID2	2 *(20)*	1 *(10)*	1
BAP1	2 *(20)*	0	1
CAMTA1	2 *(20)*	1 *(10)*	1
CLTC	2 *(20)*	0	1
DICER1	2 *(20)*	0	1
EML4	2 *(20)*	0	1
EPS15	2 *(20)*	0	1
GNAS	2 *(20)*	0	1
KDM5A	2 *(20)*	1 *(10*)	1
KDR	2 *(20)*	0	1
MET	2 *(20)*	0	1
MYH11	2 *(20)*	0	1
NIN	2 *(20)*	0	1
NUMA1	2 *(20)*	0	1
NUP98	2 *(20)*	0	1
PCSK7	2 *(20)*	3 *(30)*	1
PDGFRB	2 *(20)*	1 *(10)*	1

Number of mutated samples *n* = 10, (%). The third column lists the reported mutation frequencies in TCGA breast cancer [1]

*MET* metastasis, *PT* primary tumor, *TCGA* the cancer genome atlas

Both single-nucleotide variants (SNVs), copy-number variants (CNVs) and loss-of-heterozygosity (LOH) patterns support a finding of pronounced heterogeneity both within and between patients (Additional file 4: Figure S1a-b).

## Ti/Tv

The average Ti/Tv ratio for the heterozygous SNPs for each exome in cohort 1 was 2.7 (2.6–2.8) and for exome wide variant positions 3.37 and 7.7 in the primary tumor samples and the metastatic samples respectively (*p* < 0.05). The high Ti/Tv ratios in especially metastatic samples is likely a result of the predominance of C > T/G > A, which can be seen among the AKAP mutations (Tables 1 and 2). These substitutions are enriched in a majority of cancers and could be associated with the age at cancer diagnosis as well as adjuvant chemotherapy using alkylating drugs or adjuvant radiotherapy [25]. The Ti/Tv ratio are further known to be elevated in breast cancer, were a bias towards TpC mutation hotspots as well as a gene expression related bias towards A > G transitions compared to T > C transitions in coding strand and have been reported [26]. Specifically in breast cancer, mutational signatures clearly shows a predominance of C > T mutations, with possible associations to the ageing processes and APOBEC-induced mutagenesis [25, 27]. Interestingly, we have discovered a significant enrichment of the APOBEC related mutational signature among the metastatic tumors in cohort 2 (Ullah et al. submitted), which is in support of the high level of Ti/Tv ratio in metastatic lesions of cohort 1. A similar, enrichment of the APOBEC signature in metastatic breast cancer has also been reported by others [28].

### Mutations in the AKAP gene family are enriched in metastases

While investigating the list of recurrently mutated cancer genes we found that *AKAP9* carried an unexpected number of both silent and nonsilent mutations in the metastatic lesions. Because of the previously reported implication of *AKAP9* in breast- and other cancers, the analysis was extended to include all members of the AKAP gene family. We found a striking enrichment of mutations in the family of A-kinase anchoring proteins (AKAPs) in metastatic samples (*p* < 0.02; Fisher’s exact test) compared with primary lesions. Altogether nonsynonymous coding mutations were found in seven of the AKAP family members *AKAP5, AKAP6, AKAP8, AKAP9, AKAP10, AKAP12, AKAP13* (Table 3). Out of the ten patients in the first cohort, five (50%) carried mutations in AKAP genes in only metastatic tissue (four) or in only the primary lesion (one). Out of the total nine AKAP mutations recorded, eight were found uniquely in the metastatic lesions, indicating that these mutations have emerged in a subclone during late primary tumor evolution. None of the “metastatic-unique” mutations could be found in the corresponding primary tumors, even at low allele frequency levels.

**Table 3:** List of AKAP nonsynonymous somatic mutations in cohort 1

Gene	Position	CytoBand	Mutation	Exon	A.A substitution	Protein region	SIFT Prediction	Primary VAF	Metastasis VAF	Patient
AKAP1										
AKAP2										
AKAP3										
AKAP4										
AKAP5	chr14:64935761	14q23	G > A	2/2	Asp217Asn	NA	damaging	5.2%	0%	P2
AKAP6	chr14:33293693	14q12	G > A	13/14	Gly2225Glu	NA	tolerated	0%	17%	P1
AKAP7										
AKAP8	chr19:15484018	19q13.12	G > A	1/11	Gln169X	MCM2 binding domain	nonsense	0%	15%	P4
AKAP9	chr7:91708964	7q21.2	G > A	31/50	Ser2518Asn	close to PKA-R domain	tolerated	0%	9%	P1
AKAP10	chr17:19861659	17p11.2	A > G	4/15	Leu182Pro	RGS1 binding domain	damaging	0%	16%	P1
AKAP11										
AKAP12	chr6:151670403 chr6:151671474	6q25.1	G > A G > A	4/5 4/5	Gly293Arg Ala650Thr	EGFR interaction domain EGFR interaction domain	damaging damaging	0% 0%	22% 15%	P5 P10
AKAP13	chr15:86124141 chr15:86122728	15q25.3	C > T G > A	7/15 7/15	Gln948X Asp477Asn	NA close to PKA-RII domain	nonsense damaging	0% 0%	10% 16%	P4 P5
AKAP14										

**Fig. 2:**
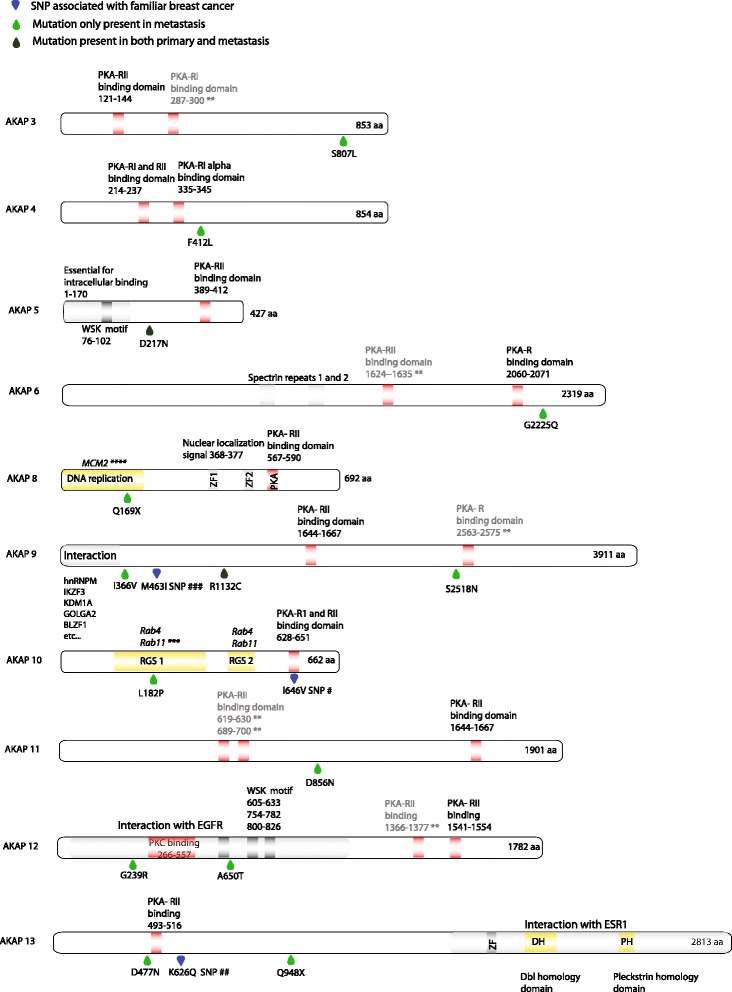
Gene view of AKAP mutations and AKAP protein regions and domains. Drop symbols indicating location of amino acid exchange light green: mutation present only in metastasis; dark green: mutation present in both primary tumor and corresponding metastasis; blue: SNP reported association with familiar breast cancer [46, 48, 66]. PKA-RI/II: protein kinase A regulatory subunit I/II; WSK: short conserved WXSXK motif in protein kinase A binding proteins (AKAPs); ZF: Zinc finger repeats; MCM2: minichromosome maintenance complex 2 [33]; DH: Dbl homology domain (RhoGEF); PH: Pleckstrin homology domain; Protein kinase interacting domains are provided from uniprotKB with additional domains from [32]**. EGFR interacting domain from [63]

*VAF* variant allele frequency; Protein region: Approximate relation to functional domains (see Fig. 2 for details). No tumor cell fraction was available for cohort 1. SIFT prediction

We next wanted to investigate this interesting mutation pattern in an independent cohort available in our lab. This cohort consists of 20 patients with paired primary breast tumor and multiple metastatic sites and is comparable regarding tumor grade, treatment and survival (Additional file 1: Table S1). Out of the 20 additional patients, four (20%) carried mutations in AKAP genes (*AKAP3, AKAP4, AKAP9, AKAP11*) in both primary tumor and metastatic tissue or in only the metastatic lesion (Table 4). In total four nonsynonymous mutations were recorded, two of these were only found in the corresponding metastatic lesion. The enrichment of mutations in metastases was not significant in cohort 2 (Fisher’s exact test), possibly because of the small size of the cohort. However, in the two patients (patients 7 and 19) having a low VAF in the primary tumors there was an increase of allele frequency in the metastatic lesions. The same increase was seen in two of the patients having multiple relapses from different time points (patient 19 and 8), indicating clonal selection. In all four patients the tumor cell fraction was over 80% in the metastatic samples. In patient 19 however, tumor cell content was as low as 20% in the primary tumor, possibly leading to an underestimation of variant allele frequency. This highlights the possible role of AKAP deregulation in the oncogenic and metastatic setting discussed in [29–31].

**Table 4:** List of AKAP nonsynonymous somatic mutations in cohort 2

Gene	Position	CytoBand	Mutation	Exon	A.A substitution	Protein region	SIFT Prediction	Primary VAF	Axillary lymph VAF	Metastasis 1 VAF	Metastasis 2 VAF	Patient
AKAP1												
AKAP2												
AKAP3	chr12:4725047	12p13.32	G > A	5/5	Ser807Leu	NA	damaging	2%	–	13%	41%, 22%^a^	pat 19
AKAP4	chrX:49958130	Xp11.22	A > G	5/6	Phe412Leu	NA	damaging	0%	0%	22%^b^	26%	pat 8
AKAP5												
AKAP6												
AKAP7												
AKAP8												
AKAP9	chr7:91630327	7q21.2	A > G	8/50	Ile366Val	close to bindning domain	tolerated	4%	–	24%, 36%^a^		pat 7
AKAP10												
AKAP11	chr13:42875448	13q14.11	G > A	8/13	Asp856Asn	NA	tolerated	0%	–	8%		pat 12
AKAP12												
AKAP13												
AKAP14												

Tumor cell fraction was determined by a clinical pathologist using blinded sample IDs

^a^multiple blocks from same sample

^b^locoregional metastasis

The protein structures of mutated AKAPs are summarized in Fig. 2 including the functional protein kinase interaction domains (PKA-RI/II, PKC) from the UniprotKB database and [32]. None of the reported mutations were located within the PKA-binding domains although metastatic mutations in *AKAP9* (S2518 N) and *AKAP13* (D477N) were located within a 20 a.a from the domain. *AKAP10* mutation L182P is located within one of the regulator of G-protein signaling (RGS1) motif [33]. Interestingly, the *AKAP8* (Q169X) mutation is located within the MCM2 binding region (see discussion), and the two *AKAP12* mutations were found in a proposed EGFR interacting domain [34].

### AKAP cytogenetics

Several copy number variations (CNV) in regions containing AKAP genes were detected. *AKAP7* and *AKAP12*, both located on chromosome 6 at 130.5 and 151 Mbp respectively, were deleted in both primary tumor and metastasis in two of the patients (P1, P5). In patient P2 the *AKAP7* and *AKAP12* deletion was aquired in the metastasis (Table 5). One patient carried deletions in both primary tumor and metastasis in a chromosome 13 region containing *AKAP11* (P1) and two additional patients seemed to have aquired the deletion in the metastasis (P3 and P8). In patient P4 *AKAP8* (chr 19) was amplified in the metastasis but also had aquired a deleterious nonsense mutation. The same patient also showed a focal amplification in chromosome 17 involving *AKAP1*.

**Table 5:** Copy number variations (CNVs) in cohorts 1 and 2 at AKAP containing loci

		P1		P2		P3		P4		P5		P8		# Amp in cohort 2		# Del in cohort 2	
Gene	Chr region	Primary	Metastasis	Primary	Metastasis	Primary	Metastasis	Primary	Metastasis	Primary	Metastasis	Primary	Metastasis	Primary	Metastasis	Primary	Metastasis
AKAP1	17q22								amp					10	11		
AKAP5	14q23						del									0	1
AKAP6	14q12						del							4	5		
AKAP7	6q23.2	del	del		del					del	del			1	4	0	2
AKAP8	19q13.12								amp					6	9		1
AKAP11	13q14.11	del	del				del						del	0	2		
AKAP12	6q25.1	del	del		del					del	del			0	2	2	3

CNV in cohort 1 were determined by calculating the normalized read coverage at each SNP position for paired tumor and germline samples, then taking the log_2_ of the ratio of these numbers and recentering to zero (see also Additional file 4: Figure S1 and Additional file 3: Figure S2)

The second cohort showed similar CNV patterns for *AKAP1* and *AKAP12*. *AKAP1* was amplified in both primary and metastasis in ten of the 20 patients. Interestingly, *AKAP1* amplifications was the most frequent mutation in the Breast Invasive Carcinoma TCGA data set altered in 8% of the tumors (*n* = 1098), data from cbioportal.org (Additional file 7: Figure S4b).

*AKAP12* was deleted in both primary and corresponding metastasis in two patients and in one patient the 6q25.1 deletion was aquired in the metastatic lesion. Loss of *AKAP12* has been shown to increase cancer incidence and metastatic spread in several cancers including prostate [35, 36] and breast cancer [37].

### AKAP gene expression

**Fig. 3:**
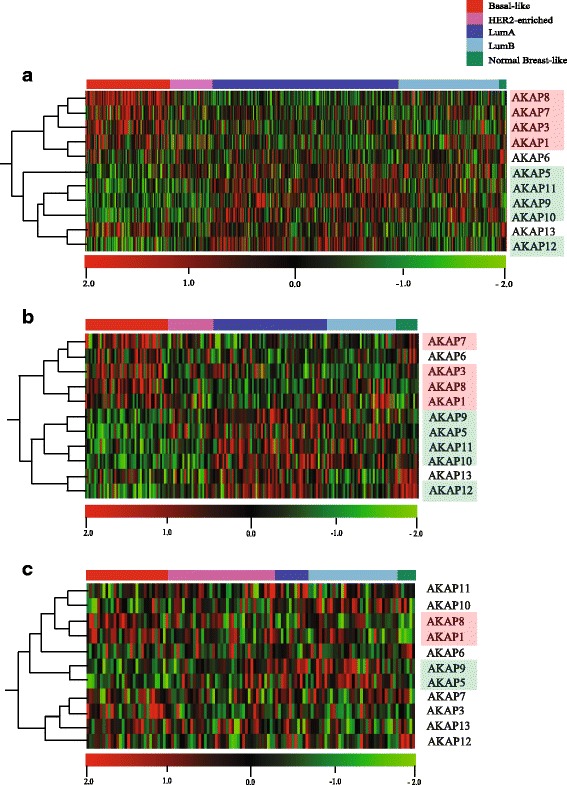
Expression profiles of the AKAP gene family in three different cohorts, tumors ordered by PAM50 subtype, genes are orderad by hierarchial clustering. **a** TCGA RNAseq expression profiles from primary tumors (*n* = 522). Genes are ordered by hierarchical clustering *p* < 0.0001 by ANOVA; q < 0.000072. Intrinsic subtype calls for the TCGA data were taken from the original manuscript [1]. **b** Risk Cohort microarray expression profiles from primary breast cancer tumors (*n* = 182). **c** Cohort 1 microarray expression profiles from metastatic lesions (*n* = 120)

As some of the AKAP members are reported with altered gene expression levels in the literature [38, 39] we wanted to further explore the gene expression of AKAP gene family in the setting of breast cancer using The Cancer Genome Atlas (TCGA) dataset (*n* = 522). Interestingly, a significant gene expression pattern of AKAPs was noted relative to tumor PAM50 subtypes whereby *AKAP1, 3, 7* and *8* were highly expressed in basal-like subtypes and lower expressed in the other subtypes (*p* < 0.001 Basal vs. other subgroups, ANOVA with post-hoc Tukey, Additional file 8: Figure S5a and Fig. 3a). Conversely, *AKAP5, 9, 10, 11* and *12* all showed lower expression in the basal-like subgroup (*p* < 0.001, ANOVA with post-hoc Tukey Additional file 8: Figure S5b and Fig. 3a). We further investigated the gene expression of the same AKAP gene sets in primary tumors from an independent nested case-control cohort, consisting of 182 patients stratified by metastatic risk with. A similar AKAP expression pattern was found (*p* < 0.001, Basal vs. other subgroups, ANOVA with post-hoc Tukey, Additional file 8: Figure S5c, d and Fig. 3b). Finally, in the full-sized TEX cohort 1 with metastatic lesions (*n* = 120), the pattern was less distinct, however, both AKAP1 and AKAP8 as well as AKAP5 ad AKAP9 showed a relative expression in the basal-like group consistent with the other cohorts (Additional file 8: Figure S5e, f and Fig. 3c).

## Discussion

In this paper we report data from the exomes of primary and metastatic lesions in breast cancer. Initially we used DNA from a small subset of patients that were part of a larger study cohort. In order to obtain sufficient DNA for exome capture we used WGA for all samples including germline. The risk of introducing false positive mutations using WGA prior sequence capture was investigated by Hasmats et al. [40] who described that the majority of false mutations were found in low coverage areas (< 10 reads), indicating that caution should be taken, both of allelic drop-out as well as low coverage regions due to problematic sequences. We addressed the issues of both false positive mutations and false negative discovery rates (Additional file 6: Table S3) and found low frequences of both, except for three of the metastatic samples in cohort 1 with high levels of false negative rates, presumably due to low starting concentrations and allelic drop out during amplification. We could not detect any other sources of variability due to different sample sources in cohort 1. We have used two different cohorts obtained at different time points as well as different sources of tumor material using two different but comparable bioinformatic pipelines. While cohort 1 was used for initial findings, cohort 2 was used for validation. Using two different cohorts, separate statistics as well as separate analytic pipelines is a methodological strength and supports our findings regarding AKAP mutations. In cohort 1, we report a large increase in number of mutations for some patients. We also found a low proportion of shared mutations between primary and metastatic tumor tissue. While the average increase in mutations in metastatic tissue was less pronounced in cohort 2 we confirm a higher mutational load in metastatic tumors. The contribution of APOBEC related mutagenesis has been revealed to be increased in metastases and might impact the mutational load in metastatic tissues. Low percentage of overlapping mutations is affected by tumor heterogeneity. Up to 70% of tumor heterogeneity both within a primary tumor but also between primary and metastasis have been reported in breast cancer and, with even higher discordance in other cancers [41]. Changes in the mutational processes and the inherent tumor heterogeneity could both lead to a low number of shared mutations.

Here, we report for the first time that somatic mutations in the AKAP gene family occur in breast cancer and are enriched in metastatic lesions. Studies including both primary breast cancer tumors and their corresponding metastatic lesions are still rare but Lefebvre et al. report the mutational profiles from metastases from over 200 patients using TCGA data as primary tumor reference [28]. In this study, mutations in the AKAP gene family are well represented with one or more AKAP mutations being present in 30 out of 216 patients (14%) being compared. In total 42 nonsynonymous mutations are present in 9 different AKAP genes, supporting our findings.

A-kinase anchoring proteins (AKAPs) are members of a protein family acting as anchors for Protein Kinase A (PKA) by specifically associating PKA regulatory subunits RI and RII to cellular organelles and directing its active signal transduction spatially and temporally via cyclic AMP [17]. This functionality has implications for both cell motility and cell proliferation. Studies show that an altered balance between subunit RI and RII has implications for tumorgenesis in prostate [42], colon [43, 44] and in addition a prognostic value in breast cancers [45]. Overexpression of subunit RI tends to increase proliferative mechanisms and is elevated in malignant lesions, whereas the RII counterpart tends to upregulate apoptotic pathways, downregulate proliferative genes and shows a decreased expression in malignant cells.

In line with the function of PKA in tumorgenesis, several of the AKAP members have been associated with cancer development and metastatic spread. Although only *AKAP9* has been previously reported as a recurrently mutated gene in COSMIC (Catalogue of Somatic Mutations in Cancer) database, several members of the AKAP family have been found to be associated with both oncogenic and tumor suppressing functions in several cancers including breast cancer. Specific polymorphisms in *AKAP10* and in particular in combination with a SNP in *AKAP13* were shown to be associated with increased risk for familial breast cancer [46]. The same polymorphism in *AKAP10 (Ile646Val)* (Fig. 2) was also found to be associated with colorectal cancer risk [47]. Additionally, specific polymorphisms in *AKAP9* are associated with increased risk in familial breast cancers [48]. *AKAP12* expression is down-regulated in various cancers summarized in [29] including breast cancer [37] and have been suggested as a suppressor of invasiveness in prostate cancer through inhibition of the PKC-Raff/MEK/ERK pathway as well as inhibition of VEGF-mediated neovascularization [49]. Here, two mutations in *AKAP12* were present in the EGFR-binding domain (Fig. 3), indicating a possible function in the deregulation of EGFR signaling pathways present in malignant cells [50]. Furthermore, the down regulation of *AKAP12* is often associated with promoter hypermethylation or loss of its locus 6q24-25.2 [51]. Based on this, *AKAP12* has been suggested to have a tumor suppressing function [49]. *AKAP12* and *AKAP11* was also shown to be essential for endothelial barrier function, the latter by linking cAMP signalling to adherens proteins such as VE-cadherin and β-catenin [52]. Similarly, *AKAP5* have been shown to localize with E-cadherin and β-catenin in epithelial adherens junctions stabilizing F-actin [53]. Furthermore, overexpression of AKAP5 was shown to reduce proliferation and hyperplasia in smooth muscle cells [54]. *AKAP9* has been shown to be essential for microtubuli dynamics at adherens junctions [55] and recently [56], demonstrated that in mice with *AKAP9* deficient T-cells, antigen- dependent activation of the T-cell by TCR recycling is impaired, suggesting a possible role in tumor cell immune surveillance (authors comment).

Taken together, *AKAP 5, 9, 11* and *12* all seem to be involved in various aspects of epithelial integrity, presenting them as potential players in cell migration and metastatic mechanisms. In our data, loss of the *AKAP12* locus occurs in six out of 30 patients. In four of these, the loss is detected in metastasis only. *AKAP5* was deleted in two of 20 patients, both in which the lesion was found uniquely in the metastasis (Table 4). Interestingly, *AKAP5*, *AKAP9, AKAP11* and *AKAP12* is expressed at lower levels in the basal-like and, less prominently in HER2-enriched subtypes (Fig. 3a-b), the breast cancer subtypes with highest risk of recurrence [57].

*AKAP13*, also named lymphoblast crisis oncogene (*AKAP-LBC)* binds, and is negatively regulated by the metastasis suppressor protein NDKA encoded by the *NM23* gene [31]. Disruption of *AKAP13* mediated anchoring of PKA decreased carcinoma cell line migration in vitro [58]. Importantly, overexpression of the NM23-H2 isoform was shown to downregulate nuclear receptor peroxisome proliferator-activated receptor δ (PPAR-δ) in human cholangiocarcinoma cells [59]. Recently it was shown [60] that *AKAP13* is essential for tamoxifen resistence induced by PKA dependent phosphorylation of ERαS305 through its direct association via ERα-binding domain and PKA. One of the mutations in *AKAP13* (D477N) found in this study, is indeed located in the PKA-RII binding subunit of *AKAP13* (Fig. 2).

*AKAP4* expression is detected at high rate in various breast cancer tumors and has been suggested as a biomarker for breast and prostate cancer [38]. Specifically *AKAP3* have been shown to play a role in cell migration and invasion in ovarian cancer [39]. *AKAP1* was shown to be required for cAMP-dependent PKA mediated apoptosis in colorectal cancer cells upon IGF1R inhibition [61]. *AKAP8* is a nuclear localized protein suggested to play a role in both DNA replication, cell cycle progression and chromatin condensation [29, 62] and also interacts with minichromosome maintenance protein 2 (MCM2). Disruption of the AKAP8-MCM2 interaction abolished DNA replication in HeLa cells [63]. In our study the *AKAP8* mutation in the metastatic lesion was located within the MCM2-binding domain (Fig. 2).

Interestingly, in line with the findings in our study, somatic mutations in *AKAP8* and *AKAP9* were found in multiple metastases in a patient with renal cell carcinoma but were both absent in the primary tumor [64]. Similarly, an *AKAP9* mutation was detected in circulating tumor DNA upon relapse, but not in the primary tumor or in blood samples prior disease progression [65]. These findings further underscore the metastasis-specific enrichment of AKAP mutations. AKAP mutations are highly prevalent in most primary cancers available in The Cancer Genome Atlas data accessed through CBioportal including CNV and point mutations (Additional file 7: Figure S4a). Lung squamous carcinoma has the highest mutation frequency with over 50% of the samples having alterations in any of AKAP genes 1–14, including 20% point mutations. In primary breast cancer almost 30% have alterations including 10% point mutations. Among these, *AKAP1* and *AKAP11* are altered in > 10%, including gene expression alterations. *AKAP3, 7, 8, 9, 10* and 13 are all altered in > 5% (Additional file 7: Figure S4b).

Our findings reveal that although present in 10% (3 of 30) primary tumors, AKAP mutations are much more prevalent (40%) in the metastatic lesions (12 of 30), indicating a clonal selection of certain malignant subpopulations during the metastatic process.

In summary, we report that several members of the A-kinase anchoring protein family (AKAPs) are mutated in metastatic breast cancer lesions. We further describe that the AKAP gene family is differently expressed in both primary and metastatic tumors and that basal-like tumors show an altered AKAP expression profile dividing the AKAPs in one highly expressed group; *AKAP1, AKAP3, AKAP7, AKAP8* and one low group; *AKAP5, AKAP9, AKAP10, AKAP11, AKAP12.* Much of previously reported functions of these individual AKAPs points towards that this AKAP clusters indeed could reflect true metastatic potential. The reduced expression of genes involved in stabilization of adherence proteins (*AKAP5, 9, 11* and *12*) as well as increase expression of genes associated with poor prognosis and proliferation (*AKAP3* and *AKAP8*) strongly underscores the involvement of AKAP genes during tumor growth and dissemination.

## Conclusions

Several studies have reported individual AKAP genes to be associated with cancer risk and specifically metastatic relapses but no study has so far demonstrated the presence of somatic genetic alterations in clinical metastatic samples. Here, we demonstrate that somatic mutations occurs in a multitude of the members of the AKAP family in breast cancer tissue, and that several of the mutations are acquired or enriched in the metastatic corresponding metastatic tumors. Several of these mutations do occur in functionally relevant regions. Further, we demonstrate that AKAP gene expression follows a subtype specific pattern that was confirmed in three independent cohorts, pinpointing a subset of the AKAP members to be interesting targets for further investigation. While conclusions regarding functionality of mutations or significance of differential AKAP gene expression, cannot be drawn here, our findings presents a novel example of convergent mutations towards a gene family with many reported implications in cancer disease. Further studies will be needed to investigate the functional role of individual AKAP genes in cancer and the implication of differential expression in breast cancer.

## Additional files

Additional file 1: **Table S1.** Patient table cohort 2. The study material includes 20 patients and was collected at Karolinska University Hospital between the years 2000 and 2011. The following inclusion criteria were applied; metastatic adenocarcinoma; detailed clinical data available; axillary and distant metastasis available; required amount of paraffin embedded tissue. The study was approved by the Ethics committee at the Karolinska Institute. (PDF 637 kb)

Additional file 2: **Table S2.** DNA concentrations. Complete list of DNA concentrations after extraction from tumor- or normal tissues and blood. For Cohort 1 total input amounts to WGA and recovered DNA amounts after is listed. (PDF 248 kb)

Additional file 3: **Figure S2.** Mean coverage of whole-exome sequencing in a) cohort 1 and b) cohort 2. b) Exome capture and sequencing was performed by SeqWright Genomic Services (GE Healthcare, Houston, USA). Briefly, exome capture was performed using Sure Select XT2 Human All ExonV5 (Agilent Technologies) according to manufacturers instructions. Paired end sequencing was performed on Illumina HiSeq 2500. Raw sequencing reads were quality and adapter trimmed using trim_galore, where the first 13 bases of Illumina adapter were used and stringency parameter was set to 2. Reads having lengths less than 70 after trimming were filtered out with their paired mates. Trimmed reads were aligned to the human reference genome build hg19 using bwa-mem with default parameters. The aligned reads were marked for duplicates by Picard, realigned around known indels and base-quality recalibrated by GATK. Somatic single nucleotide variants (SNVs) were detected by Mutect with the high-confidence mode. Somatic short indels were detected by Varscan2 with minimum variant allele frequency of 0.05. Copy number alterations were detected by ADTEx/Ascat. All pipelines and analyses were run using Anduril, a workflow framework for scientific data analysis. (PDF 915 kb)

Additional file 4: **Figures S1a and S1b.** Exome-wide view of mutations in metastatic breast cancer. Each panel (numbered by patient ID) shows whole-exome data from one patient in several aligned tracks, as indicated on the right. SNV: single-nucleotide variations, shown as tick marks for COSMIC recurrent cancer genes (outer tracks) and as density for all mutations (inner tracks). The central colored density gradient shows major allele concordance *P* value between primary and metastasis (50 SNP moving windows; blue: concordant; red: discordant), indicating the presence of concordant loss of heterozygosity. CNV: copy-number variation (red: deletion, blue: amplification); LOH: loss-of-heterozygosity. The Tumorscape track shows known hotspots for copy-number variation in breast cancer using data from Tumorscape Copy Number Alterations Across Multiple Cancer Types Release 1.6 (Broad Institute). CNV was determined by calculating the normalized read coverage at each SNP position for paired tumor and germline samples, then taking the log_2_ of the ratio of these numbers and recentering to zero. In Fig. 1, these measures were plotted as a moving average across ten adjacent SNPs. LOH: loss of heterozygousity) as major allele frequency for heterozygous SNPs on the vertical axis, with manually called LOH regions indicated by horizontal lines (vertical axis range 50–100%; red: significant SNP phase concordance with the paired sample, *p* < 0.01 by the binomial distribution; black: not significant (see also Additional file 3: Figure S2 a-b). Note that LOH could not be called for some samples (metastases of P1, P2, P4 and P10) because of low data quality. SNP phase concordance was determined using all SNPs in the LOH region in A and recorded the major allele of each (i.e. the ‘phase’ of the LOH region). We then tested the null hypothesis that the alleles of the corresponding SNPs in sample B was randomly distributed relative to A, with a 50% chance of concordance at each position. This would be expected if there were in fact no LOH in B, as the major allele would then be determined by the random fluctuations of read coverage. Under this model, the probability of observing *k* concordant calls among *n* SNPs is distributed according to the Binomial distribution (*k*; *n*, *p*) with *p* = 0.5. Thus we calculated the *P* value as the cumulative density of this distribution from *k* to *n* (that is, the probability of observing as many as *k* concordant calls, or more). A low *P* value (e.g. *P <* 0.01) on this test suggest that the LOH region in A was in fact also present on B, whereas a high *P* (e.g. *P >* 0.99) value suggests that there was LOH in B, but it was derived from the opposite allele compared with A. Intermediate values are expected whenever there was no LOH in sample B. The central track for each patient in Additional file 4: Figure S1 shows this *P* value. (ZIP 1670 kb)

Additional file 5: **Figures S3a and S3b.** Estimation of LOH fraction and tumor content. Estimation of LOH fraction and tumor content using a Beta-Normal mixture model. Histograms show the distribution of major allele frequencies (range: 50–100%) and red curves show the mixture model estimated from the data. The set of SNPs called in the germline sample was considered in the tumor sample (primary and metastasis independently). When genomes contain regions of LOH, the distribution will be bimodal, as illustrated in the inset (top right). The first component (closer to 50%; red in inset) represents heterozygous SNPs in regions without LOH and was modeled as a normal distribution with a mean close to 50% in a perfect sample, but will increase towards 100% as allelic dropout increases. The second component (closer to 100%; blue in inset) represents homozygous SNPs in regions with LOH, was modeled as a beta distribution with two parameters (the mean of this component should be close to 100% and increase as LOH fraction increases and decrease as with non-cancer cell contamination. The mixture distribution thus had four free parameters plus the mixture proportion, the latter representing the estimated LOH fraction of the sample. Fitting this mixture to the observations yielded estimates for LOH fraction, tumor fraction and allelic dropout rate for each sample (Additional file 3: Figure S2). Model fitting was performed using the *EstimatedDistribution* function of Mathematica 9.0 (Wolfram Research Inc.). (ZIP 421 kb)

Additional file 6: **Table S3.** Control experiment. To investigate the rate of false positive mutations as well as the loss of alleles introduced by the amplification process we designed a control experiment as follows: Genomic DNA from a healthy individual was extracted from whole blood using the PAXgene Blood DNA kit (Qiagen). The DNA was of good quality (> 58 kB fragment length) and of high concentration (> 300 ng/uL). This DNA was diluted 1:100 and 1:1000 and 6 ng respectively 0.6 ng was subjected to WGA, exome enriched, sequenced and analyzed as described in (Fig. 2a) using an unamplified sample as ‘germline’ control. In the control exome experiment with 6 ng input gDNA, we found no (zero) variant positions, indicating that the false discovery rate was negligible when the amount of starting material was in the range of 6 ng or more. In the control exome experiment with 0.6 ng input gDNA, we found 27 amino acid altering mutations that passed our SNV calling criteria. This indicates that false positive SNVs can start to appear as the amount of input material is reduced. We used this higher rate of false positives to estimate the false discovery rate (FDR) in samples with input less than 6 ng. Note that no tumor sample was amplified from less than 1.2 ng DNA. Allelic drop out or loss of heterozygosity due to biased amplification towards one of the alleles will be challenging to distinguish from true LOH. As expected, in the control exomes no LOH was detected. The fraction of called LOH positions was 0.6% and 1% of all variant positions in the 6 ng and 0.6 ng control experiments respectively, and these positions did not form continuous regions. In most tumor samples, we could clearly distinguish regions of true LOH, as blocks of SNPs with LOH calls. Outside these regions, any observed LOH could be assumed to be artefactual. We therefore determined the fraction of LOH calls in regions without signs of true LOH in the tumor samples, as a measure of false negative calls (allelic dropout). In the primary tumor samples we found 1.36% (0.1%- 8.3%) false negatives, whereas in the metastatic samples this fraction was 4.88% (0%–16.25%). However, three of the samples (metastases of patients P1, P4 and P10) were of poor quality showing a very noisy pattern of variant allele frequencies, most likely due to DNA degradation and low concentration. In these three samples the false negative discovery rate could not be calculated but could be approximated by assuming that there were no true LOH calls at all, resulting in a conservative estimate of 43–87% false negative calls in these three samples. We could not identify LOH independently in these samples, but we used the SNP phase test described in (Additional file 4: Figure S1a-b) to confirm or reject the existence of LOH alterations in the metastatic lesion concordant with the primary tumor. Although the median false negative SNV rate was less than 1%, the metastatic samples of three patients (P1, P4 and P10) suffered a rate exceeding 40%, presumably due to allelic dropout during amplification. The median false discovery rate was 1%, and did not exceed 6% for any patient. (PDF 398 kb)

Additional file 7: **Figure S4.** AKAP mutations in TCGA data. a) AKAP mutations across all cancers available through cBioPortal (TCGA, provisional). b) AKAP mutations and gene expression alterations in Breast Invasive Carcinoma, (TCGA, provisional). (PDF 753 kb)

Additional file 8: **Figure S5.** AKAP gene expression. Boxplots showing summed expression of AKAP 8,7,3,1 and AKAPs 5,11,9,10,12 gene expression within each PAM50 molecular subgroup, a-b; TCGA data, c-d; risk cohort, d-e; cohort 1. *P*-values are indicative of ANOVA followed by post-hoc Tukey for Basal vs. other subtypes individually. ***; *p* ≤ 0.001, **; *p* ≤ 0.01. (PDF 586 kb)

## Abbreviations

AKAP: A-kinase anchoring proteins
CNV: Copy number variation
EGFR: Epithelial growth factor receptor
ER: Estrogen receptor
LOH: Loss of heterozygosity
MDA: Multiple displacement amplification
PKA: Protein kinase A
PR: Progesteron receptor
SNV: Single nucleotide variation
TCGA: The cancer genome atlas
VEGFR: Vascular endothelial growth factor receptor

## References

[1] Koboldt DC, Fulton RS, Mclellan MD, Schmidt H, Kalicki-Veizer J, Mcmichael JF (2012). Comprehensive molecular portraits of human breast tumours. Nature.

[2] Stephens PJ, Tarpey PS, Davies H, Van Loo P, Greenman C, Wedge DC (2012). The landscape of cancer genes and mutational processes in breast cancer. Nature.

[3] Sjöblom T, Jones S, Wood LD, Parsons DW, Lin J, Barber TD (2006). The consensus coding sequences of human breast and colorectal cancers. Science.

[4] Lindstrom LS, Karlsson E, Wilking UM, Johansson U, Hartman J, Lidbrink EK (2012). Clinically used breast cancer markers such as estrogen receptor, progesterone receptor, and human epidermal growth factor receptor 2 are unstable throughout tumor progression. J Clin Oncol.

[5] Foukakis T, Åström G, Lindström L, Hatschek T, Bergh J (2012). When to order a biopsy to characterise a metastatic relapse in breast cancer. Ann Oncol.

[6] Amir E, Ooi WS, Simmons C, Kahn H, Christakis M, Popovic S (2008). Discordance between receptor status in primary and metastatic breast cancer: an exploratory study of bone and bone marrow biopsies. Clin Oncol (R Coll Radiol).

[7] Almendro V, Kim HJ, Cheng Y-K, Gonen M, Itzkovitz S, Argani P (2014). Genetic and phenotypic diversity in breast tumor metastases. Cancer Res.

[8] Torres L, Ribeiro FR, Pandis N, Andersen JA, Heim S, Teixeira MR (2007). Intratumor genomic heterogeneity in breast cancer with clonal divergence between primary carcinomas and lymph node metastases. Breast Cancer Res Treat.

[9] Shah SP, Morin RD, Khattra J, Prentice L, Pugh T, Burleigh A (2009). Mutational evolution in a lobular breast tumour profiled at single nucleotide resolution. Nature.

[10] de Bruin EC, McGranahan N, Mitter R, Salm M, Wedge DC, Yates L (2014). Spatial and temporal diversity in genomic instability processes defines lung cancer evolution. Science.

[11] Goswami RS, Patel KP, Singh RR, Meric-Bernstam F, Kopetz ES, Subbiah V (2015). Hotspot mutation panel testing reveals Clonal evolution in a study of 265 paired primary and metastatic tumors. Clin Cancer Res.

[12] Gundem G, Van Loo P, Kremeyer B, Alexandrov LB, JMC T, Papaemmanuil E (2015). The evolutionary history of lethal metastatic prostate cancer. Nature.

[13] Brastianos PK, Carter SL, Santagata S, Cahill DP, Taylor-Weiner A, Jones RT (2015). Genomic characterization of brain metastases reveals branched evolution and potential therapeutic targets. Cancer Discov.

[14] Toy W, Shen Y, Won H, Green B, Sakr RA, Will M (2013). ESR1 ligand-binding domain mutations in hormone-resistant breast cancer. Nat Genet.

[15] Robinson DR, Wu Y-M, Vats P, Su F, Lonigro RJ, Cao X (2013). Activating ESR1 mutations in hormone-resistant metastatic breast cancer. Nat Genet.

[16] Jeselsohn R, Yelensky R, Buchwalter G, Frampton G, Meric-Bernstam F, Gonzalez-Angulo AM (2014). Emergence of constitutively active estrogen receptor-α mutations in pretreated advanced estrogen receptor-positive breast cancer. Clin Cancer Res.

[17] Colledge M, Scott JD (1999). AKAPs: from structure to function. Trends Cell Biol.

[18] Hatschek T, Carlsson L, Einbeigi Z, Lidbrink E, Linderholm B, Lindh B (2011). Individually tailored treatment with epirubicin and paclitaxel with or without capecitabine as first-line chemotherapy in metastatic breast cancer: a randomized multicenter trial. Breast Cancer Res Treat.

[19] Koboldt DC, Zhang Q, Larson DE, Shen D, McLellan MD, Lin L (2012). VarScan 2: somatic mutation and copy number alteration discovery in cancer by exome sequencing. Genome Res.

[20] Cibulskis K, Lawrence MS, Carter SL, Sivachenko A, Jaffe D, Sougnez C (2013). Sensitive detection of somatic point mutations in impure and heterogeneous cancer samples. Nat Biotechnol.

[21] Amarasinghe KC, Li J, Hunter SM, Ryland GL, Cowin PA, Campbell IG (2014). Inferring copy number and genotype in tumour exome data. BMC Genomics.

[22] Ovaska K, Laakso M, Haapa-Paananen S, Louhimo R, Chen P, Aittomäki V (2010). Large-scale data integration framework provides a comprehensive view on glioblastoma multiforme. Genome Med.

[23] Tobin NP, Harrell JC, Lövrot J, Brage SE, Stolt MF, Carlsson L (2014). Molecular subtype and tumor characteristics of breast cancer metastases as assessed by gene expression significantly influence patient post-relapse survival. Ann Oncol.

[24] Cunha SI, Bocci M, Lövrot J, Eleftheriou N, Roswall P, Cordero E (2015). Endothelial ALK1 is a therapeutic target to block metastatic dissemination of breast cancer. Cancer Res.

[25] Alexandrov LB, Nik-Zainal S, Wedge DC, Aparicio SAJR, Behjati S, Biankin AV (2013). Signatures of mutational processes in human cancer. Nature.

[26] Rubin AF, Green P (2009). Mutation patterns in cancer genomes. Proc Natl Acad Sci U S A.

[27] Roberts SA, Lawrence MS, Klimczak LJ, Grimm SA, Fargo D, Stojanov P (2013). ng.2702. Nat Genet.

[28] Lefebvre C, Bachelot T, Filleron T, Pedrero M, Campone M, Soria J-C (2016). Mutational profile of metastatic breast cancers: a retrospective analysis. PLoS Med.

[29] Han B, Poppinga WJ, Schmidt M (2015). Scaffolding during the cell cycle by A-kinase anchoring proteins. Pflugers Arch.

[30] Chowdhury S, Howell GM, Rajput A, Teggart CA, Brattain LE, Weber HR (2011). Identification of a novel TGFβ/PKA signaling transduceome in mediating control of cell survival and metastasis in colon cancer. PLoS One.

[31] Iwashita S, Fujii M, Mukai H, Ono Y, Miyamoto M (2004). Lbc proto-oncogene product binds to and could be negatively regulated by metastasis suppressor nm23-H2. Biochem Biophys Res Commun.

[32] Burgers PP, van der Heyden MAG, Kok B, Heck AJR, Scholten A (2015). A systematic evaluation of protein Kinase A–A-Kinase anchoring protein interaction motifs. Biochemistry.

[33] Eggers CT, Schafer JC, Goldenring JR, Taylor SS (2009). D-AKAP2 interacts with Rab4 and Rab11 through its RGS domains and regulates transferrin receptor recycling. J Biol Chem.

[34] Deribe YL, Wild P, Chandrashaker A, Curak J, Schmidt MHH, Kalaidzidis Y (2009). Regulation of epidermal growth factor receptor trafficking by lysine deacetylase HDAC6. Sci Signal.

[35] Su B, Bu Y, Engelberg D, Gelman IH (2010). SSeCKS/Gravin/AKAP12 inhibits cancer cell invasiveness and chemotaxis by suppressing a protein kinase C- Raf/MEK/ERK pathway. J Biol Chem.

[36] Akakura S, Huang C, Nelson PJ, Foster B, Gelman IH (2008). Loss of the SSeCKS/Gravin/AKAP12 gene results in prostatic hyperplasia. Cancer Res.

[37] Perou CM, Sørlie T, Eisen MB, van de Rijn M, Jeffrey SS, Rees CA (2000). Molecular portraits of human breast tumours. Nature.

[38] Saini S, Jagadish N, Gupta A, Bhatnagar A, Suri A. A novel cancer testis antigen, a-Kinase anchor protein 4 (AKAP4) is a potential biomarker for breast cancer. Miller TW, editor. PLoS One. 2013;8(2):e57095. 10.1371/journal.pone.0057095 PMC3579772 23451156

[39] Sharma S, Qian F, Keitz B, Driscoll D, Scanlan MJ, Skipper J (2005). A-kinase anchoring protein 3 messenger RNA expression correlates with poor prognosis in epithelial ovarian cancer. Gynecol Oncol.

[40] Hasmats J, Gréen H, Orear C, Validire P, Huss M, Käller M (2014). Assessment of whole genome amplification for sequence capture and massively parallel sequencing. PLoS One.

[41] McGranahan N, Swanton C (2017). Clonal heterogeneity and tumor evolution: past, present, and the future. Cell.

[42] Neary CL, Nesterova M, Cho YS, Cheadle C, Becker KG, Cho-Chung YS (2004). Protein kinase A isozyme switching: eliciting differential cAMP signaling and tumor reversion. Oncogene.

[43] Carlson CC, Smithers SL, Yeh KA, Burnham LL, Dransfield DT (1999). Protein kinase A regulatory subunits in colon cancer. Neoplasia.

[44] Hu Z-Y, Liu Y-P, Xie L-Y, Wang X-Y, Yang F, Chen S-Y (2016). AKAP-9 promotes colorectal cancer development by regulating Cdc42 interacting protein 4. Biochim Biophys Acta.

[45] Miller WR (2002). Regulatory subunits of PKA and breast cancer. Ann N Y Acad Sci.

[46] Wirtenberger M, Schmutzhard J, Hemminki K, Meindl A, Sutter C, Schmutzler RK (2007). The functional genetic variant Ile646Val located in the kinase binding domain of the A-kinase anchoring protein 10 is associated with familial breast cancer. Carcinogenesis.

[47] Wang M, Zhang D, Wang R, Rui Y, Zhou J, Wang R (2013). A-Kinase anchoring proteins 10 expression in relation to 2073A/G polymorphism and tumor progression in patients with colorectal cancer. Pathol Oncol Res.

[48] Frank B, Wiestler M, Kropp S, Hemminki K, Spurdle AB, Sutter C (2008). Association of a Common AKAP9 variant with breast cancer risk: a collaborative analysis. J Natl Cancer Inst.

[49] Gelman IH (2012). Suppression of tumor and metastasis progression through the scaffolding functions of SSeCKS/Gravin/AKAP12. Cancer Metastasis Rev.

[50] Tomas A, Futter CE, Eden ER (2014). EGF receptor trafficking: consequences for signaling and cancer. Trends Cell Biol.

[51] Jin Z, Cheng Y, Gu W, Zheng Y, Sato F, Mori Y (2009). A multicenter, double-blinded validation study of methylation biomarkers for progression prediction in Barrett's esophagus. Cancer Res.

[52] Radeva MY, Kugelmann D, Spindler V, Waschke J. PKA compartmentalization via AKAP220 and AKAP12 contributes to endothelial barrier regulation. Komarova Y, editor. PLoS One. 2014;9(9):e106733. 10.1371/journal.pone.0106733 PMC4154725 25188285

[53] Gorski JA, Gomez LL, Scott JD, Dell'Acqua ML (2005). Association of an A-kinase-anchoring protein signaling scaffold with cadherin adhesion molecules in neurons and epithelial cells. Mol Biol Cell.

[54] Indolfi C, Stabile E, Coppola C, Gallo A, Perrino C, Allevato G (2001). Membrane-bound protein kinase A inhibits smooth muscle cell proliferation in vitro and in vivo by amplifying cAMP-protein kinase A signals. Circ Res.

[55] Sehrawat S, Ernandez T, Cullere X, Takahashi M, Ono Y, Komarova Y (2011). AKAP9 regulation of microtubule dynamics promotes Epac1-induced endothelial barrier properties. Blood.

[56] Herter JM, Grabie N, Cullere X, Azcutia V, Rosetti F, Bennett P (2015). AKAP9 regulates activation-induced retention of T lymphocytes at sites of inflammation. Nat Commun.

[57] Voduc KD, Cheang MCU, Tyldesley S, Gelmon K, Nielsen TO, Kennecke H (2010). Breast cancer subtypes and the risk of local and regional relapse. J Clin Oncol.

[58] Paulucci-Holthauzen AA, Vergara LA, Bellot LJ, Canton D, Scott JD, O'Connor KL (2009). Spatial distribution of protein Kinase a activity during cell migration is mediated by A-kinase anchoring protein AKAP Lbc. J Biol Chem.

[59] He F, York JP, Burroughs SG, Qin L, Xia J, Chen D (2015). Recruited metastasis suppressor NM23-H2 attenuates expression and activity of peroxisome proliferator-activated receptor δ (PPARδ) in human cholangiocarcinoma. Dig Liver Dis.

[60] Bentin Toaldo C, Alexi X, Beelen K, Kok M, Hauptmann M, Jansen M (2015). Protein Kinase A-induced tamoxifen resistance is mediated by anchoring protein AKAP13. BMC Cancer.

[61] Hedrick ED, Agarwal E, Leiphrakpam PD, Haferbier KL, Brattain MG, Chowdhury S (2013). Differential PKA activation and AKAP association determines cell fate in cancer cells. J Mol Signal.

[62] Akileswaran L, Taraska JW, Sayer JA, Gettemy JM, Coghlan VM (2001). A-kinase-anchoring protein AKAP95 is targeted to the nuclear matrix and associates with p68 RNA helicase. J Biol Chem.

[63] Eide T, Tasken KA, Carlson C, Williams G, Jahnsen T, Tasken K (2003). Protein Kinase A-anchoring protein AKAP95 interacts with MCM2, a regulator of DNA replication. J Biol Chem.

[64] Gerlinger M, Rowan AJ, Horswell S, Larkin J, Endesfelder D, Gronroos E (2012). Intratumor heterogeneity and branched evolution revealed by multiregion sequencing. N Engl J Med.

[65] Jansen MP, Martens JW, Helmijr JC, Beaufort CM, van Marion R, Krol NM (2016). Cell-free DNA mutations as biomarkers in breast cancer patients receiving tamoxifen. Oncotarget.

[66] Wirtenberger M, Tchatchou S, Hemminki K, Klaes R, Schmutzler RK, Bermejo JL (2006). Association of genetic variants in the rho guanine nucleotide exchange factor AKAP13 with familial breast cancer. Carcinogenesis.

