# Supplementary material for: Exome sequencing of primary breast cancers with paired metastatic lesions reveals metastasis-enriched mutations in the A-kinase anchoring protein family (AKAPs)
Source: BMC Cancer. 2018 Feb 12;18:174. doi: 10.1186/s12885-018-4021-6 (PMC5810006; doi:10.1186/s12885-018-4021-6)
Supplement: Supplementary file 1 — Table S1. Patient table cohort 2. The study material includes 20 patients and was collected at Karolinska University Hospital between the years 2000 and 2011. The following inclusion criteria were applied; metastatic adenocarcinoma; detailed clinical data available; axillary and distant metastasis available; required amount of paraffin embedded tissue. The study was approved by the Ethics committee at the Karolinska Institute. (PDF 637 kb) [file 12885_2018_4021_MOESM1_ESM.pdf]

Table S1. Patient table cohort 2

Patient ID	Sample	Elston Grade	Tum size (mm)	ER	PR	HER2	NeoAdj	AdjCT	AdjHT	AdjRT	Relapse site	Time to Relapse*	Relapse to death**
1	*Primary Tumor*	*3*	*21*	-	-	-	-	+	-	+			
	Metastasis 1			-	-	*NA*					lung	*19*	
	Metastasis 2			-	-	*NA*					liver	*49*	35
2	*Primary Tumor*	*3*	*20*	-	-	*NA*	-	-	-	-			
	Lymph node										lymph		
	Metastasis 1			-	-	*NA*					colon	*57*	1
3	*Primary Tumor*	*3*	*55*	-	-	+	*CT*	+	-	+			
	Lymph node										lymph		
	Metastasis 1			-	-	*NA*					bone	*11*	2
4	*Primary Tumor*	*3*	*20*	-	-	*NA*	-	-	-	-			
	Metastasis 2			*NA*	*NA*	*NA*					uterus	*74*	
	Metastasis 3			-	-	*NA*					brain	*93*	
	Metastasis 4			+	-	*NA*					colon	*97*	60
5	*Primary Tumor*	*3*	*25*	+	-	*NA*	*HT*	-	+	+			
	Metastasis 1			-	-	*NA*					bone	*31*	
	Metastasis 2			*NA*	*NA*	*NA*					bone	*49*	28
6	*Primary Tumor*	*2*	*20*	+	+	+	-	+	+	+			48
7	*(Primary Tumor)*			*NA*	*NA*	-	-	+	+	+			
	Local relapse	*3*		+	-	+					chest wall	*54*	
	Metastasis 1			+	-	+					skin	*64*	29
8	*Primary Tumor*	*3*	*45*	-	-	-	-	+	+	+			
	Lymph node												
	Local rel	*3*		+	-	+					skin local relapse	*49*	
	Metastasis 1			+	-	*NA*					bone	*75*	29
9	*Primary Tumor*	*3*	*18*	+	-	+	-	+	+	+			
	Metastasis 1			-	-	-					brain	*26*	23
10	*Primary Tumor*	*3*	*45*	-	-	+	-	+	-	+			
	Lymph node										lymph		
	Metastasis 2			-	-	*NA*					skin	*54*	60
11	*Primary Tumor*	*3*	*35*	-	+	*NA*	-	+	+	+			
	Metastasis 1			-	-	*NA*					brain	*24*	10
12	*Primary Tumor*	*3*	*55*	-	-	*NA*	-	-	-	-			
	Local relapse			-	-	*NA*					axillary lymph	*19*	
	Metastasis 1										bone	*39*	41
13	*(Primary Tumor)*			*NA*	*NA*	*NA*	-	NA	NA	+			
	Local relapse	*3*		+	+	*NA*					breast	*84*	
	Local relapse										lymph	*120*	79
	Metastasis 1			*NA*	*NA*	*NA*					bone	*156*	
14	*Primary Tumor*	*3*	*30*	+	+	+	-	+	+	+			
	Lymph node										lymph		
	Metastasis 1			*NA*	*NA*	*NA*					cerebellum	*26*	83
15	*Primary Tumor*	*NA*	*10*	+	*NA*	*NA*	-	+	+	+			
	Lymph node										lymph		
	Metastasis 1			*NA*	*NA*	*NA*					liver	*116*	26
16	*(Primary Tumor)*	*3*	*35*	-	-	+	-	+	-	-			
	Local relapse	*3*		-	-	+					breast	*12*	
	Metastasis 1			*NA*	*NA*	*NA*					skin	*16*	27
17	*Primary Tumor*	*3*	*18*	+	+	*NA*	-	-	+	+			
	Lymph node										lymph		
	Metastasis 1			-	-	*NA*					brain	*75*	5
18	*Primary Tumor*	*2*	*20*	+	+	-	-	+	+	+			
	Lymph node										lymph		
	Metastasis 2			+	+	+					skin	*87*	47
19	*Primary Tumor*	*2*	*20*	+	+	*NA*	*CT*	+	+	+			
	Metastasis 3			-	-	+					brain	*73*	
	Metastasis 5			*NA*	*NA*	*NA*					brain	*88*	79
20	*Primary Tumor*	*2*	*20*	-	+	+	*CT*	-	+	-			
	Metastasis 2	*2*		+	+	+					ovary	*262*	
