# Supplementary material for: Exome sequencing of primary breast cancers with paired metastatic lesions reveals metastasis-enriched mutations in the A-kinase anchoring protein family (AKAPs)
Source: BMC Cancer. 2018 Feb 12;18:174. doi: 10.1186/s12885-018-4021-6 (PMC5810006; doi:10.1186/s12885-018-4021-6)
Supplement: Supplementary file 2 — Table S2. DNA concentrations. Complete list of DNA concentrations after extraction from tumor- or normal tissues and blood. For Cohort 1 total input amounts to WGA and recovered DNA amounts after is listed. (PDF 248 kb) [file 12885_2018_4021_MOESM2_ESM.pdf]

Table S2. DNA concentrations

		ID	Extracted DNA Concetration (ng/ul)	Total input to WGA (ng)	Total amount DNA after WGA (ug)
Cohort 1	Primary tumors (fresh frozen)	P1	233.55	10	4.3
		P2	239.55	10	3.9
		P3	15.13	10	8.3
		P4	23.05	10	11.0
		P5	545.98	10	26.5
		P6	280.21	10	6.0
		P7	55.91	10	8.8
		P8	1.3	6.5	30.8
		P9	422.5	88.6	30.4
		P10	292.5	59.2	34.8
	Metastasis (fine needle aspiratons)	P1	5.9	1.29	7.1
		P2	9.6	4.12	6.3
		P3	19.5	4.46	19.4
		P4	7.6	2.5	8.4
		P5	3	4.49	12.3
		P6	54	78.4	17.9
		P7	117	54.2	15.6
		P8	1.3	6.5	17.3
		P9	687.5	89.4	71.1
		P10	4.5	3.8	12.5
	Blood (fresh frozen)	P1	916	10	32.3
		P2	400	10	39.7
		P3	36	10	36.0
		P4	280	10	30.6
		P5	132	10	42.8
		P6	92	10	32.2
		P7	41	10	31.7
		P8	47.2	94.4	27.2
		P9	320	102	10.2
		P10	315	74.6	49.9
Control experiment	normal DNA	1:1	313	626	-
		1:100		6	120.0
		1:1000		0.6	86.16
Cohort 2	Primary tumors (FFPE)	1	149.5		
		2	247.6		
		3	82.6		
		4	102.5		
		5	289.8		
		6	44.0		
		7	31.2		
		8	89.8		
		9	376.9		
		10	112.7		
		11	60.3		
		12	210.7		
		13	124.3		
		14	97.3		
		15	37.2		
		16	182.4		
		17	48.0		
		18	83.0		
		19	251.0		
		20	43.5		
	Metastastases (FFPE)	1 lung	357.9		
		1 liver	54.5		
		2 lymph	150.7		
		2 colon	254.5		
		3 lymph	77.2		
		3 bone	27.6		
		4 uterus	33.4		
		4 brain	176.1		
		4 colon	29.4		
		5 bone	283.1		
		5 bone	178.0		
		7 skin	13.0		
		8 skin	19.9		
		8 bone	509.0		
		9 brain	227.6		
		10 lymph	229.1		
		10 skin	124.8		
		11 brain	21.9		
		12 lymph	31.3		
		12 bone	60.7		
		13 lymph	102.7		
		13 bone	101.0		
		14 lymph	122.4		
		14 cerebellum	119.1		
		15 lymph	86.5		
		16 skin	89.3		
		17 lymph	132.0		
		17 brain	50.7		
		18 lymph	83.3		
		18 skin	470.5		
		19 brain	142.7		
		19 brain	71.3		
	(Fresh DNA)	20 ovary	16.7		
	Control DNA (FFPE)	1	29.2		
		2	193.3		
		3	93.4		
		4	23.4		
		5	17.3		
		6	161.4		
		7	37.3		
		8	55.6		
		9	4.3		
		10	98.9		
		11	13.3		
		12	145.1		
		13	334.9		
		14	57.8		
		15	168.7		
		16	25.4		
		17	95.9		
		18	20.0		
		19	81.9		
		20	38.0		
