# Supplementary figures and images for: Exome sequencing of primary breast cancers with paired metastatic lesions reveals metastasis-enriched mutations in the A-kinase anchoring protein family (AKAPs)
Source: BMC Cancer. 2018 Feb 12;18:174. doi: 10.1186/s12885-018-4021-6 (PMC5810006; doi:10.1186/s12885-018-4021-6)

Figure S2

a)

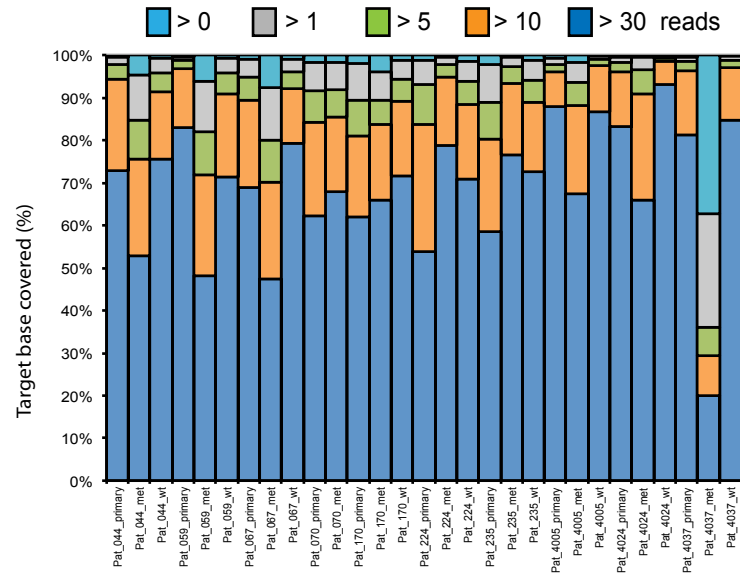

b)

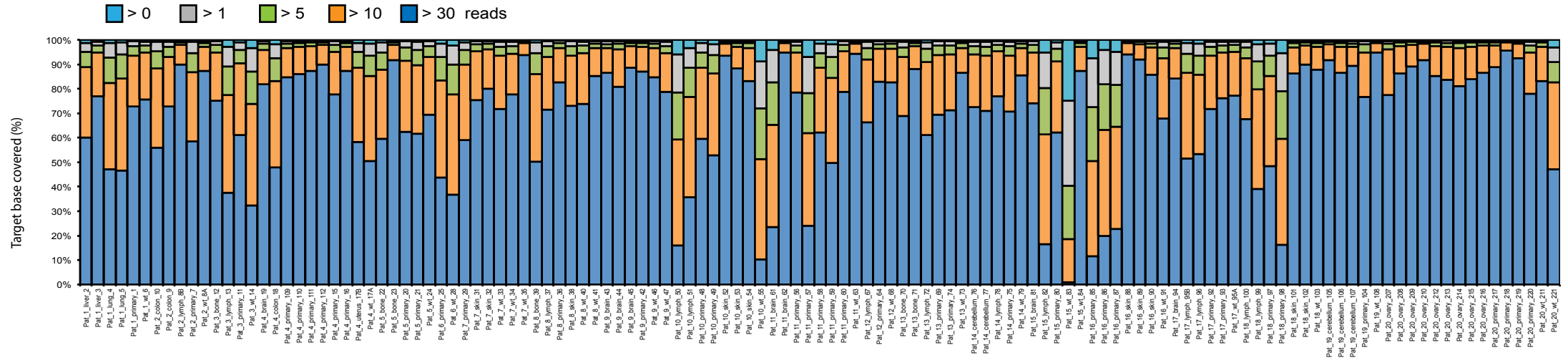

Supplement: Supplementary file 3 — Figure S2. Mean coverage of whole-exome sequencing in a) cohort 1 and b) cohort 2. b) Exome capture and sequencing was performed by SeqWright Genomic Services (GE Healthcare, Houston, USA). Briefly, exome capture was performed using Sure Select XT2 Human All ExonV5 (Agilent Technologies) according to manufacturers instructions. Paired end sequencing was performed on Illumina HiSeq 2500. Raw sequencing reads were quality and adapter trimmed using trim_galore, where the first 13 bases of Illumina adapter were used and stringency parameter was set to 2. Reads having lengths less than 70 after trimming were filtered out with their paired mates. Trimmed reads were aligned to the human reference genome build hg19 using bwa-mem with default parameters. The aligned reads were marked for duplicates by Picard, realigned around known indels and base-quality recalibrated by GATK. Somatic single nucleotide variants (SNVs) were detected by Mutect with the high-confidence mode. Somatic short indels were detected by Varscan2 with minimum variant allele frequency of 0.05. Copy number alterations were detected by ADTEx/Ascat. All pipelines and analyses were run using Anduril, a workflow framework for scientific data analysis. (PDF 915 kb) [file 12885_2018_4021_MOESM3_ESM.pdf]

Figure S1a

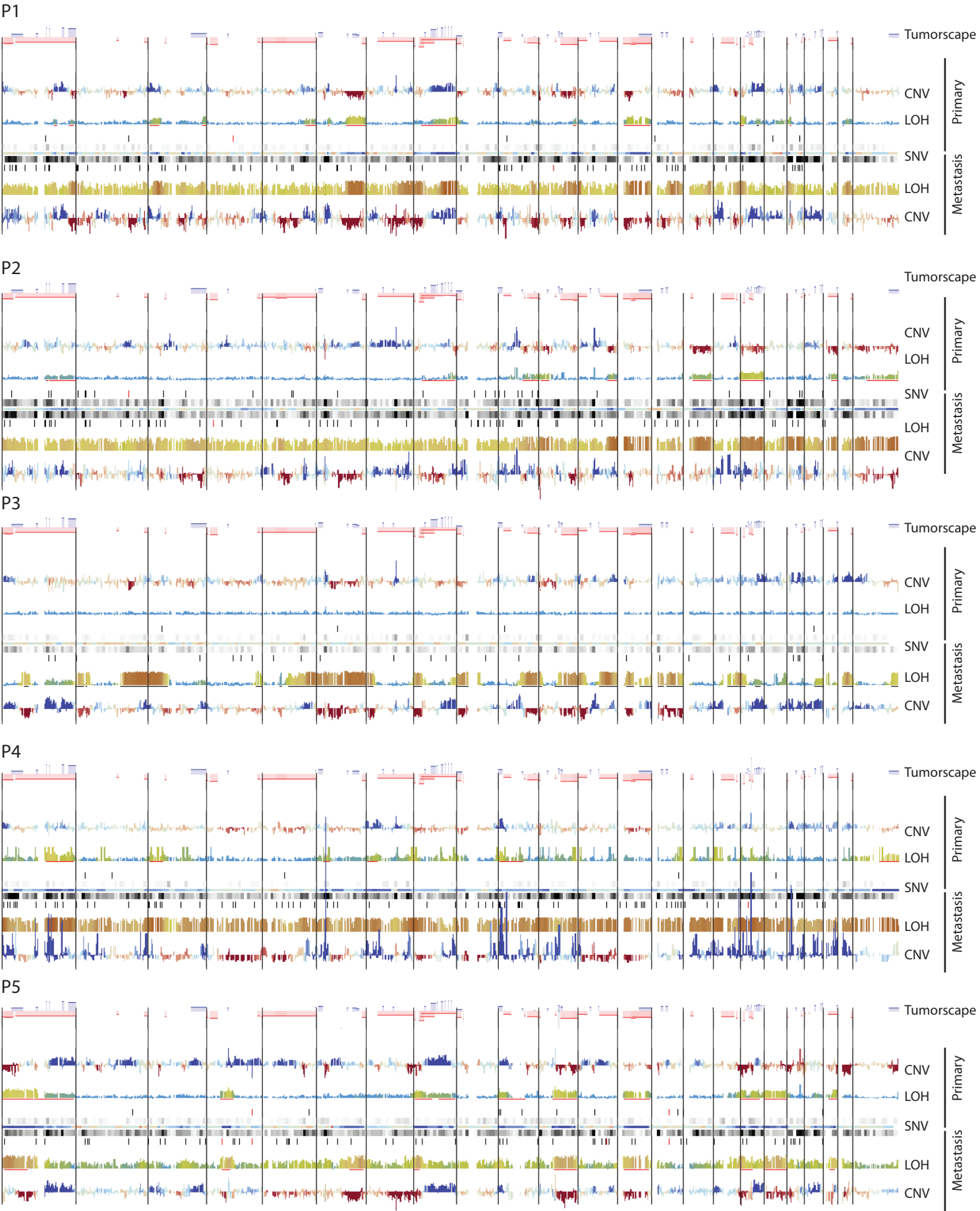

Supplement: Supplementary file 4 — Figures S1a and S1b. Exome-wide view of mutations in metastatic breast cancer. Each panel (numbered by patient ID) shows whole-exome data from one patient in several aligned tracks, as indicated on the right. SNV: single-nucleotide variations, shown as tick marks for COSMIC recurrent cancer genes (outer tracks) and as density for all mutations (inner tracks). The central colored density gradient shows major allele concordance P value between primary and metastasis (50 SNP moving windows; blue: concordant; red: discordant), indicating the presence of concordant loss of heterozygosity. CNV: copy-number variation (red: deletion, blue: amplification); LOH: loss-of-heterozygosity. The Tumorscape track shows known hotspots for copy-number variation in breast cancer using data from Tumorscape Copy Number Alterations Across Multiple Cancer Types Release 1.6 (Broad Institute). CNV was determined by calculating the normalized read coverage at each SNP position for paired tumor and germline samples, then taking the log2 of the ratio of these numbers and recentering to zero. In Fig. 1, these measures were plotted as a moving average across ten adjacent SNPs. LOH: loss of heterozygousity) as major allele frequency for heterozygous SNPs on the vertical axis, with manually called LOH regions indicated by horizontal lines (vertical axis range 50–100%; red: significant SNP phase concordance with the paired sample, p < 0.01 by the binomial distribution; black: not significant (see also Additional file 3: Figure S2 a-b). Note that LOH could not be called for some samples (metastases of P1, P2, P4 and P10) because of low data quality. SNP phase concordance was determined using all SNPs in the LOH region in A and recorded the major allele of each (i.e. the ‘phase’ of the LOH region). We then tested the null hypothesis that the alleles of the corresponding SNPs in sample B was randomly distributed relative to A, with a 50% chance of concordance at each position. This would be expected if [file 12885_2018_4021_MOESM4_ESM.zip › S Fig1aR4.pdf]

Figure S1b

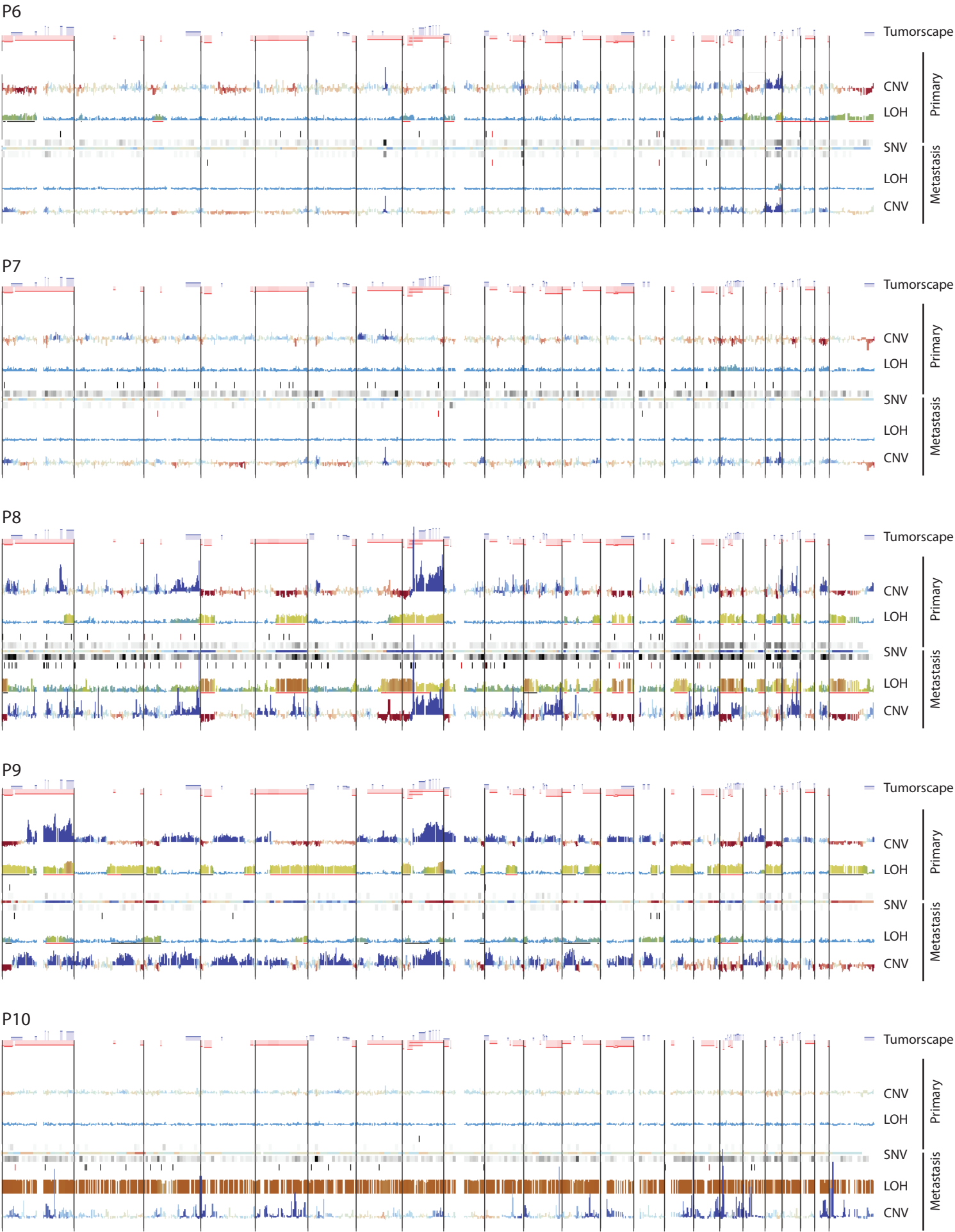

Supplement: Supplementary file 4 — Figures S1a and S1b. Exome-wide view of mutations in metastatic breast cancer. Each panel (numbered by patient ID) shows whole-exome data from one patient in several aligned tracks, as indicated on the right. SNV: single-nucleotide variations, shown as tick marks for COSMIC recurrent cancer genes (outer tracks) and as density for all mutations (inner tracks). The central colored density gradient shows major allele concordance P value between primary and metastasis (50 SNP moving windows; blue: concordant; red: discordant), indicating the presence of concordant loss of heterozygosity. CNV: copy-number variation (red: deletion, blue: amplification); LOH: loss-of-heterozygosity. The Tumorscape track shows known hotspots for copy-number variation in breast cancer using data from Tumorscape Copy Number Alterations Across Multiple Cancer Types Release 1.6 (Broad Institute). CNV was determined by calculating the normalized read coverage at each SNP position for paired tumor and germline samples, then taking the log2 of the ratio of these numbers and recentering to zero. In Fig. 1, these measures were plotted as a moving average across ten adjacent SNPs. LOH: loss of heterozygousity) as major allele frequency for heterozygous SNPs on the vertical axis, with manually called LOH regions indicated by horizontal lines (vertical axis range 50–100%; red: significant SNP phase concordance with the paired sample, p < 0.01 by the binomial distribution; black: not significant (see also Additional file 3: Figure S2 a-b). Note that LOH could not be called for some samples (metastases of P1, P2, P4 and P10) because of low data quality. SNP phase concordance was determined using all SNPs in the LOH region in A and recorded the major allele of each (i.e. the ‘phase’ of the LOH region). We then tested the null hypothesis that the alleles of the corresponding SNPs in sample B was randomly distributed relative to A, with a 50% chance of concordance at each position. This would be expected if [file 12885_2018_4021_MOESM4_ESM.zip › S Fig1bR4.pdf]

Figure S3a

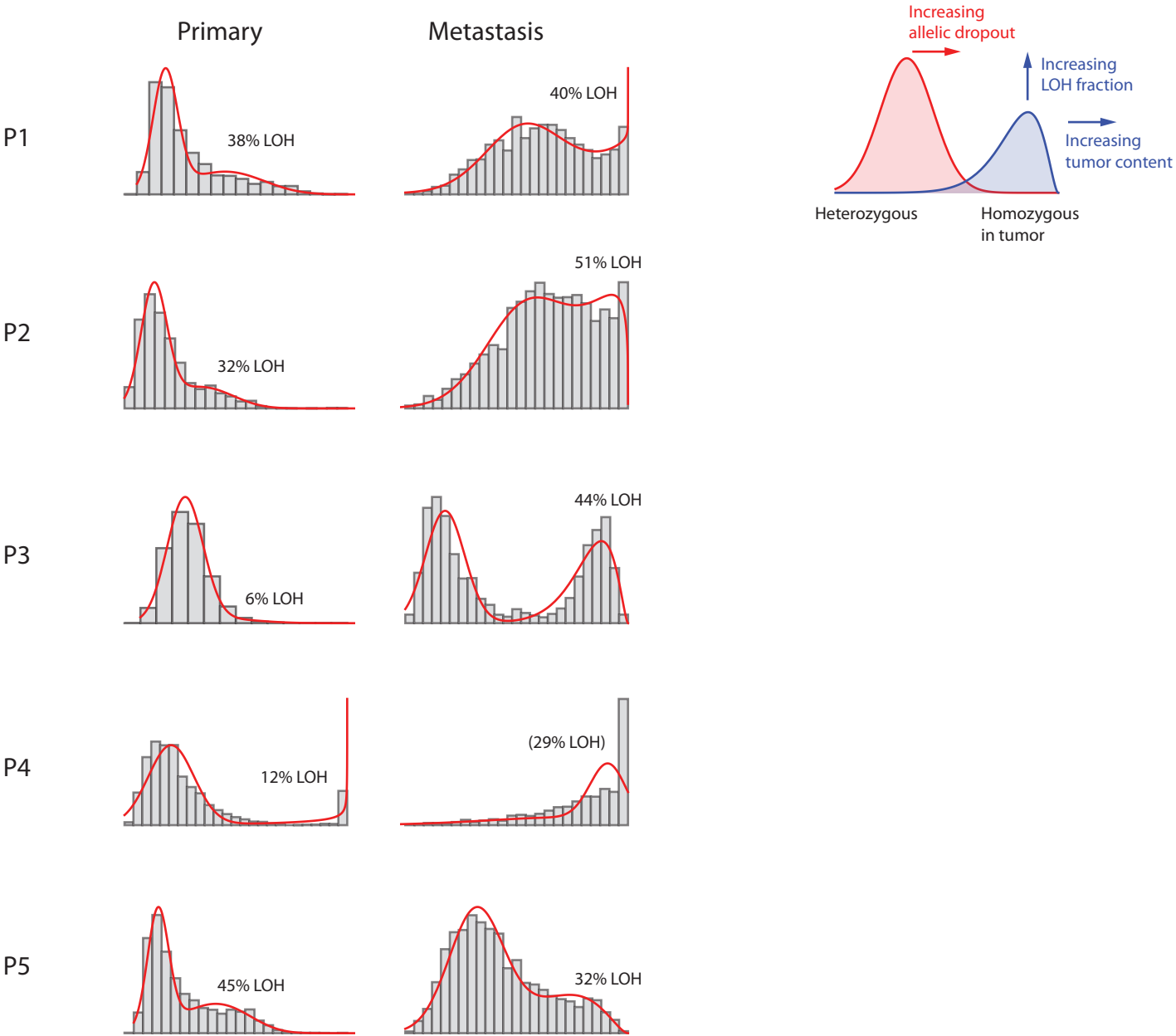

Supplement: Supplementary file 5 — Figures S3a and S3b. Estimation of LOH fraction and tumor content. Estimation of LOH fraction and tumor content using a Beta-Normal mixture model. Histograms show the distribution of major allele frequencies (range: 50–100%) and red curves show the mixture model estimated from the data. The set of SNPs called in the germline sample was considered in the tumor sample (primary and metastasis independently). When genomes contain regions of LOH, the distribution will be bimodal, as illustrated in the inset (top right). The first component (closer to 50%; red in inset) represents heterozygous SNPs in regions without LOH and was modeled as a normal distribution with a mean close to 50% in a perfect sample, but will increase towards 100% as allelic dropout increases. The second component (closer to 100%; blue in inset) represents homozygous SNPs in regions with LOH, was modeled as a beta distribution with two parameters (the mean of this component should be close to 100% and increase as LOH fraction increases and decrease as with non-cancer cell contamination. The mixture distribution thus had four free parameters plus the mixture proportion, the latter representing the estimated LOH fraction of the sample. Fitting this mixture to the observations yielded estimates for LOH fraction, tumor fraction and allelic dropout rate for each sample (Additional file 3: Figure S2). Model fitting was performed using the EstimatedDistribution function of Mathematica 9.0 (Wolfram Research Inc.). (ZIP 421 kb) [file 12885_2018_4021_MOESM5_ESM.zip › S Figure_S3aR4.pdf]

Figure S3b

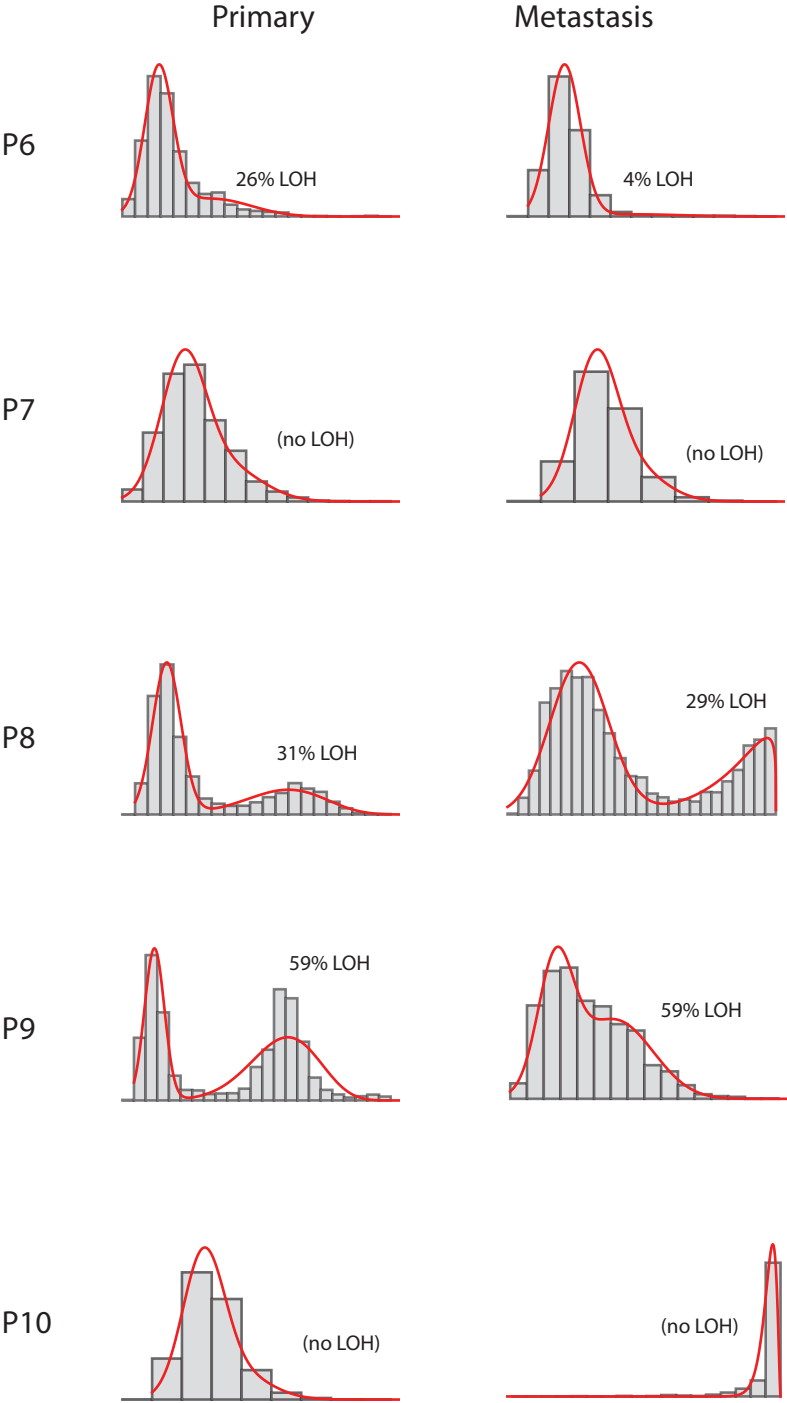

Supplement: Supplementary file 5 — Figures S3a and S3b. Estimation of LOH fraction and tumor content. Estimation of LOH fraction and tumor content using a Beta-Normal mixture model. Histograms show the distribution of major allele frequencies (range: 50–100%) and red curves show the mixture model estimated from the data. The set of SNPs called in the germline sample was considered in the tumor sample (primary and metastasis independently). When genomes contain regions of LOH, the distribution will be bimodal, as illustrated in the inset (top right). The first component (closer to 50%; red in inset) represents heterozygous SNPs in regions without LOH and was modeled as a normal distribution with a mean close to 50% in a perfect sample, but will increase towards 100% as allelic dropout increases. The second component (closer to 100%; blue in inset) represents homozygous SNPs in regions with LOH, was modeled as a beta distribution with two parameters (the mean of this component should be close to 100% and increase as LOH fraction increases and decrease as with non-cancer cell contamination. The mixture distribution thus had four free parameters plus the mixture proportion, the latter representing the estimated LOH fraction of the sample. Fitting this mixture to the observations yielded estimates for LOH fraction, tumor fraction and allelic dropout rate for each sample (Additional file 3: Figure S2). Model fitting was performed using the EstimatedDistribution function of Mathematica 9.0 (Wolfram Research Inc.). (ZIP 421 kb) [file 12885_2018_4021_MOESM5_ESM.zip › S Figure_S3bR4.pdf]

a)

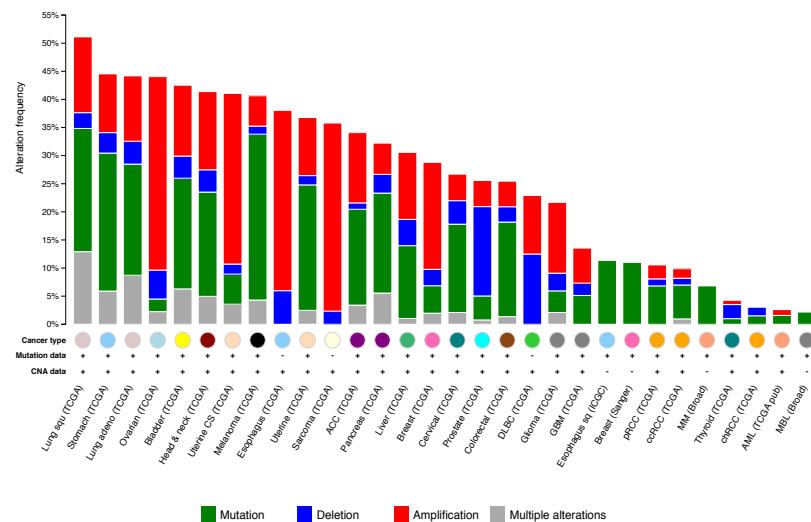

b)

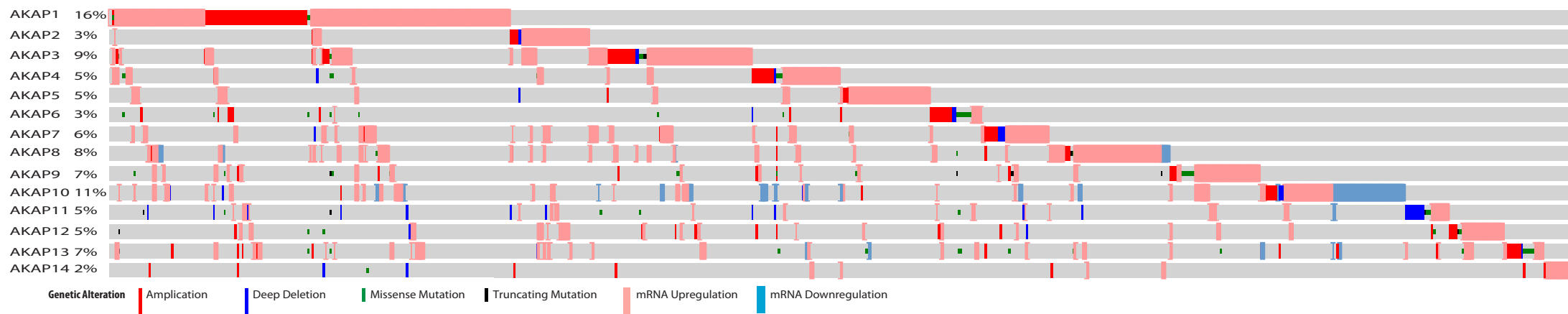

Supplement: Supplementary file 7 — Figure S4. AKAP mutations in TCGA data. a) AKAP mutations across all cancers available through cBioPortal (TCGA, provisional). b) AKAP mutations and gene expression alterations in Breast Invasive Carcinoma, (TCGA, provisional). (PDF 753 kb) [file 12885_2018_4021_MOESM7_ESM.pdf]
