# Supplementary material for: Exome sequencing of primary breast cancers with paired metastatic lesions reveals metastasis-enriched mutations in the A-kinase anchoring protein family (AKAPs)
Source: BMC Cancer. 2018 Feb 12;18:174. doi: 10.1186/s12885-018-4021-6 (PMC5810006; doi:10.1186/s12885-018-4021-6)
Supplement: Supplementary file 6 — Table S3. Control experiment. To investigate the rate of false positive mutations as well as the loss of alleles introduced by the amplification process we designed a control experiment as follows: Genomic DNA from a healthy individual was extracted from whole blood using the PAXgene Blood DNA kit (Qiagen). The DNA was of good quality (> 58 kB fragment length) and of high concentration (> 300 ng/uL). This DNA was diluted 1:100 and 1:1000 and 6 ng respectively 0.6 ng was subjected to WGA, exome enriched, sequenced and analyzed as described in (Fig. 2a) using an unamplified sample as ‘germline’ control. In the control exome experiment with 6 ng input gDNA, we found no (zero) variant positions, indicating that the false discovery rate was negligible when the amount of starting material was in the range of 6 ng or more. In the control exome experiment with 0.6 ng input gDNA, we found 27 amino acid altering mutations that passed our SNV calling criteria. This indicates that false positive SNVs can start to appear as the amount of input material is reduced. We used this higher rate of false positives to estimate the false discovery rate (FDR) in samples with input less than 6 ng. Note that no tumor sample was amplified from less than 1.2 ng DNA. Allelic drop out or loss of heterozygosity due to biased amplification towards one of the alleles will be challenging to distinguish from true LOH. As expected, in the control exomes no LOH was detected. The fraction of called LOH positions was 0.6% and 1% of all variant positions in the 6 ng and 0.6 ng control experiments respectively, and these positions did not form continuous regions. In most tumor samples, we could clearly distinguish regions of true LOH, as blocks of SNPs with LOH calls. Outside these regions, any observed LOH could be assumed to be artefactual. We therefore determined the fraction of LOH calls in regions without signs of true LOH in the tumor samples, as a measure of false negative calls (allelic dropout). [file 12885_2018_4021_MOESM6_ESM.pdf]

Table S3. Control experiment

	Average input DNA (ng)	Somatic mutations	False Discovery Rate (%)	LOH of heterozygous SNPs (%)	False LOH (%)
Germline	34.1 *(10-103)*	-	-	-	-
Primary Tumor	22.4 *(6.5-88.6)*	222	0	5.2 *(0.1-18)*	1.6 *(0.1-8.3)*
Metastasis	24.9 *(1.3-89.4)*	706	2.2 *(0-7)*	18.9 *(0-48)* *	5.7 *(0-16.2)* *
Control 6ng	6.0	0	100	1	100
Control 0.6ng	0.6	27	100	0.6	100
