# Supplementary material for: Exome sequencing of primary breast cancers with paired metastatic lesions reveals metastasis-enriched mutations in the A-kinase anchoring protein family (AKAPs)
Source: BMC Cancer. 2018 Feb 12;18:174. doi: 10.1186/s12885-018-4021-6 (PMC5810006; doi:10.1186/s12885-018-4021-6)

AKAP expression split by PAM50 subtype

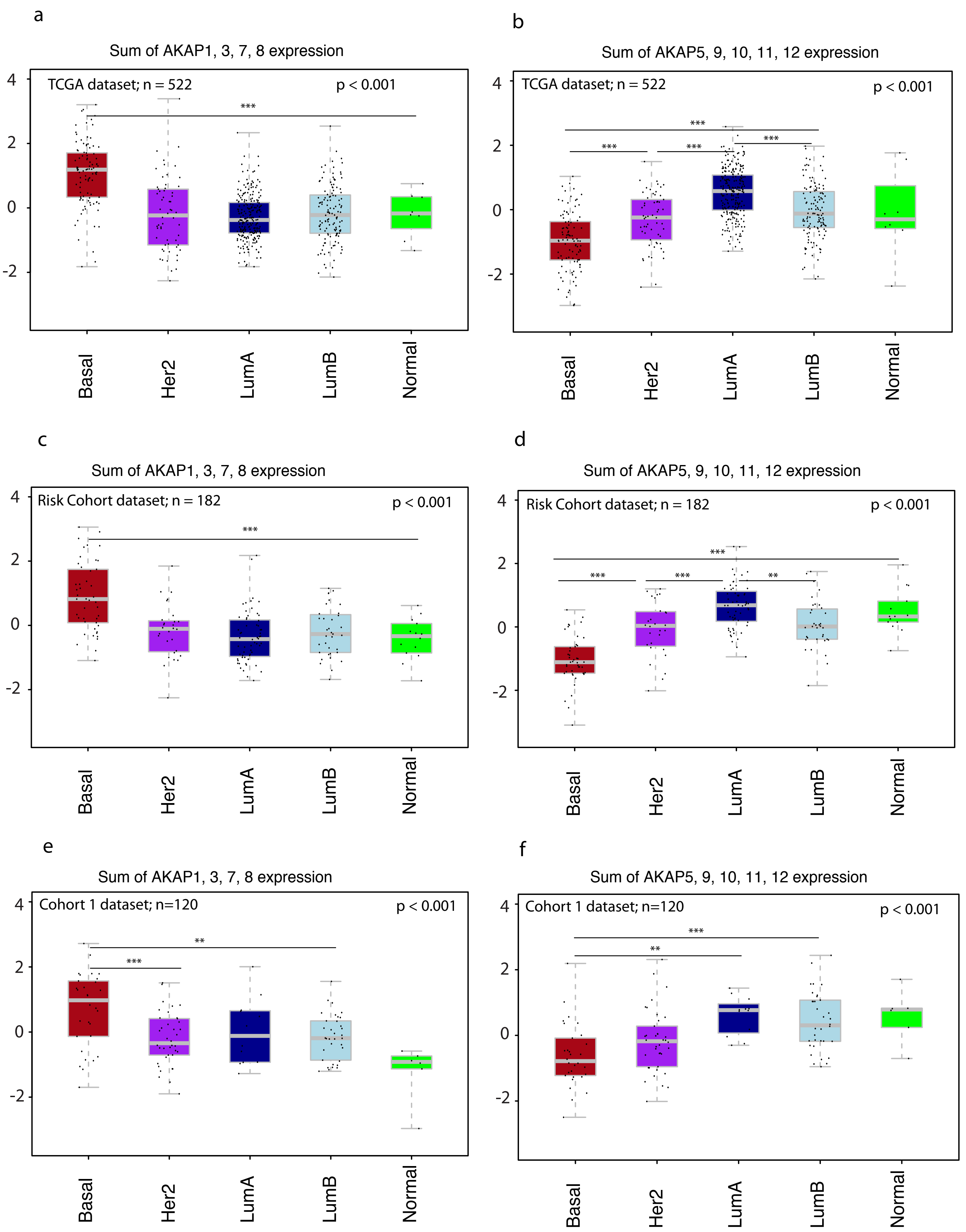

Supplement: Supplementary file 8 — Figure S5. AKAP gene expression. Boxplots showing summed expression of AKAP 8,7,3,1 and AKAPs 5,11,9,10,12 gene expression within each PAM50 molecular subgroup, a-b; TCGA data, c-d; risk cohort, d-e; cohort 1. P-values are indicative of ANOVA followed by post-hoc Tukey for Basal vs. other subtypes individually. ***; p ≤ 0.001, **; p ≤ 0.01. (PDF 586 kb) [file 12885_2018_4021_MOESM8_ESM.pdf]
